# Exploration of biomarkers associated with histone lactylation modification in spinal cord injury

**DOI:** 10.3389/fgene.2025.1609439

**Published:** 2025-07-02

**Authors:** Yisong Sun, Jie Gao, Juehua Jing

**Affiliations:** ^1^ Department of Orthopaedics, The Second Affiliated Hospital of Anhui Medical University, Hefei, China; ^2^ Department of Clinical Laboratory, The First Affiliated Hospital of Bengbu Medical University, Bengbu, China

**Keywords:** spinal cord Injury, histone lactylation modification, cluster, Biomarkers, gene expression omnibus

## Abstract

**Introduction:**

The biological roles of histone lactylation (HLA) modification-related genes (HLMRGs) in spinal cord injury (SCI) remain unclear. This study aimed to investigate the expression patterns and molecular mechanisms of HLMRGs in SCI through bioinformatics approaches.

**Methods:**

Data from GSE151371, GSE47681, and 10 HLMRGs were analyzed. Subsequently, biomarkers were identified based on receiver operating characteristic (ROC) curves, followed by logistic regression modeling and nomogram construction. Gene set enrichment analysis (GSEA) was performed to assess the functional roles of these biomarkers. Clustering analysis of samples based on biomarkers revealed distinct groups, and differentially expressed genes between these groups were analyzed. Inter-cluster comparisons were conducted to examine Hallmark pathways, HLA genes, and immune functions. Weighted gene co-expression network analysis (WGCNA) was applied to identify cluster-related module genes, which were further used for protein-protein interaction (PPI) network construction to pinpoint key proteins. Networks linking miRNAs, transcription factors (TFs), and biomarkers, as well as drug-biomarker interactions, were established. The expression of biomarkers was validated through reverse transcription-quantitative polymerase chain reaction (RT-qPCR).

**Results:**

In GSE151371, eight biomarkers (*HDAC1*, *HDAC2*, *HDAC3*, *SIRT1*, *SIRT3*, *LDHA*, *LDHB*, and *GCN5* [*KAT2A*]) exhibited area under the curve (AUC) > 0.7 and were significantly differentially expressed between SCI and control samples. These biomarkers also showed differential expression across the two identified clusters. Differential expression analysis between clusters 1 and 2 revealed enrichment in pathways such as the 'phosphatidylinositol signaling system.' Finally, a miRNA-TF-biomarker network involving the eight biomarkers was constructed, and their expression was validated by RT-qPCR. It is noteworthy that the expression of *HDAC2*, *GCN5* (*KAT2A*), *LDHA*, *HDAC3*, and *SIRT3* showed significant differences between SCI and control samples. This suggests that these genes may have potential clinical significance in SCI and warrant further validation. Additionally, by exploring their mechanisms of action in depth, they may provide important biomarkers for the early diagnosis, treatment strategy optimization, and personalized medicine of SCI, thereby advancing clinical research and drug development related to SCI.

**Conclusion:**

In summary, 8 biomarkers playing an important role in SCI were identified, providing a reference for the application of HLMRGs in SCI.

## 1 Introduction

Spinal cord injury (SCI) can lead to motor and sensory impairments below the injury site, potentially resulting in limb paralysis and urinary or fecal incontinence, significantly diminishing patients’ quality of life. Approximately 90% of SCI cases are caused by trauma, such as traffic accidents or falls (Huang and Lu, 2021). Currently, no reliable treatment options exist for patients with severe neurological damage. Most clinical interventions focus on stabilizing the patient, preventing further injury, and managing complications arising from paralysis (Eli et al., 2021; Eckert and Martin, 2017). Current treatment methods include surgical intervention (Bühren et al., 1999), drug therapy (Liu et al., 2019; Torelli et al., 2022), and hyperbaric oxygen therapy (Siglioccolo et al., 2024). Additionally, stem cell transplantation (Zipser et al., 2022) and gene therapy (Smith et al., 2022) have shown effectiveness in research and are poised for broader clinical application, offering new avenues for SCI rehabilitation. Following SCI, spinal cord nerve cells undergo apoptosis and necrosis. Due to the irreparability of nerve cells and the inhibitory effects of the injury’s local microenvironment, recovery from SCI remains challenging. Emerging treatments, particularly bone marrow mesenchymal stem cell (BMSC) transplantation and gene therapy, are showing promising results (Li et al., 2019; Cofano et al., 2019). While surgery and pharmacological interventions may partially restore nerve function in patients with SCI, numerous neurological dysfunctions often persist or exacerbate during the secondary injury phase. Recent advancements in understanding SCI pathophysiology, molecular interactions, neuroprotection, immune regulation, and neural regeneration pathways have garnered significant attention from researchers worldwide (Anjum et al., 2020). Despite these advances, effective treatment for neurological damage remains elusive. Therefore, exploring potential biomarkers and their molecular mechanisms in SCI is crucial for improving diagnostic and therapeutic approaches.

The nucleosome, the fundamental unit of chromatin, consists of an octamer of histones around which DNA is wound. Histones, as essential components of chromatin, undergo post-translational chemical modifications that serve as critical mechanisms of epigenetic regulation (Galle et al., 2022). These modifications include acetylation, butyrylation, methylation, and phosphorylation. Histone lactylation is a newly identified modification in this category. Lactic acid is converted into lactyl-CoA, which donates a lactyl group to the lysine residues on histone tails via acyltransferase, resulting in histone lactylation. The lactyl group in this modification is derived from lactic acid (Zhang et al., 2019; Lv et al., 2023; Dai et al., 2022). In central nervous system (CNS) diseases, histone lactylation plays a pivotal role in immune regulation and maintaining cellular homeostasis (Yang et al., 2025). Additionally, Alzheimer’s disease (AD), a neurodegenerative disorder of the CNS, has been linked to elevated histone lactylation levels. Increased histone lactylation in AD mouse brain samples exacerbates microglial dysfunction, while inhibition of histone lactylation improves microglial function and enhances spatial learning and memory in AD mice. SCI, another CNS disorder, shares pathological features with AD, including neuroinflammation, metabolic dysfunction, and overlapping clinical manifestations. Both SCI and AD exhibit neuroinflammatory responses, driven by phenotypic changes in microglia and macrophages, which hinder neurological recovery (Devanney et al., 2020). In AD, neurofibrillary tangles of intracellular tau protein are a hallmark, while in SCI models, tau inhibition has been shown to improve recovery by reducing neuroinflammation and oxidative stress (Che et al., 2023). Tau protein levels in cerebrospinal fluid (CSF) are also indicative of SCI severity (Roerig et al., 2013). Furthermore, emerging studies suggest that SCI may activate the C/EBPβ-AEP axis, mediating cognitive dysfunction via APP C586/Tau N368 segment diffusion, thus presenting clinical symptoms resembling AD (Wu et al., 2023). Although some studies suggest a potential link between SCI and AD, the exact nature of this relationship remains poorly understood. While histone lactylation has been shown to play a significant role in neurological diseases, its specific involvement in SCI remains unclear. The phenotypic changes in inflammatory cells induced by histone lactylation and their contribution to neuroinflammation in SCI require further investigation. Therefore, identifying biomarkers related to histone lactylation modification in SCI is essential for advancing our understanding of this pathology (Pan et al., 2022).

This study integrated transcriptomic data related to SCI, identified biomarkers associated with histone lactylation, constructed bioinformatics-based diagnostic models, and analyzed the biological functions of these biomarkers, providing novel insights for clinical diagnosis.

## 2 Methods

### 2.1 Data source

The study utilized datasets obtained from the Gene Expression Omnibus (GEO) website (https://www.ncbi.nlm.nih.gov/geo/). The training set, GSE151371 (GPL20301), included sequencing data from 38 SCI samples and 10 healthy controls. The validation set, GSE47681 (GPL1261), consisted of sequencing data from mouse spinal cord tissue, comprising nine control mice, nine mice on day 3 post-SCI, and nine mice on day 7 post-SCI. A total of 10 histone lactylation modification-related genes (HLMRGs) were analyzed: *HDAC1*, *HDAC2*, *HDAC3*, *SIRT1*, *SIRT2*, *SIRT3* (Moreno-Yruela et al., 2022), *LDHA*, *LDHB* (Yu et al., 2021); *P300* (EP300) (Zhang et al., 2019), and *GCN5* (*KAT2A*) (Wang et al., 2022).

### 2.2 Expression of HLMRGs in SCI

To assess the expression of HLMRGs in SCI, a Wilcoxon test was first applied to compare the expression between SCI and control samples in the GSE151371 dataset. Additionally, a heatmap of the expression profiles of the 10 HLMRGs across all samples was generated using the Pheatmap package (v1.0.12, https://CRAN.R-project.org/package=pheatmap). Correlations between the 10 HLMRGs were analyzed using Spearman’s correlation. The RCircos package (v1.2.2) (Zhang et al., 2013) was used to map the 10 HLMRGs to their respective chromosomes.

### 2.3 Construction and evaluation of logistic regression model

The gene expression data was log-transformed (log2 (x+1)) to stabilize variance and meet the normality assumption. Samples and genes with missing values were filtered using the na. omit () method. Logistic regression and SVM classification were applied, with regularization to prevent overfitting. The model’s performance on imbalanced data was evaluated using the area under curve (AUC)-receiver operating characteristic (ROC) curve based on assessment metrics. The ROC curves for differentially expressed HLMRGs (DE-HLMRGs) in SCI and control samples from GSE151371 were plotted using the pROC package (v1.1.1) (Robin et al., 2011). Genes with AUC greater than 0.7 were considered as potential biomarkers for constructing a logistic regression model, and the model’s performance was evaluated using the ROC curve. The createDataPartition () method was used for stratified sampling by default, ensuring that the ratio of the training and testing sets matched the original data distribution. Finally, 10-fold cross-validation was performed using trainControl to reduce the randomness of a single data split and enhance the reliability of model stability evaluation.

### 2.4 Construction and evaluation of nomogram model

Based on the identified biomarkers in the GSE151371 dataset, missing values were removed, and a logistic regression model was fitted using the lrm () function. The data distribution was then set using the datadist () function for subsequent analysis. Next, the nomogram () function from the R package rms (v1.6) (https://CRAN.R-project.org/package=rms) was used to generate a nomogram, specifying the transformation function for the predicted probabilities. The plot () function was subsequently used to visualize the nomogram to assess the clinical applicability of the model. Finally, the calibrate () function was used to perform calibration analysis, evaluating the consistency between the predicted probabilities and the actual observed probabilities. The C-statistic was used to evaluate the calibration curve, referring to the proportion of all possible positive-negative sample pairs in which the model predicted the positive samples with higher probabilities. This statistic effectively measured the model’s predictive ability and accuracy. When the C-statistic value was greater than 0.7, it indicated a stronger correlation and better performance of the model. Decision curve analysis (DCA) was performed using the rms package (v1.6) to assess whether the nomogram prediction was beneficial to patients with SCI.

### 2.5 Gene Set Enrichment Analysis (GSEA) for biomarkers

To explore the biological functions and signaling pathways associated with the biomarkers, the correlation coefficients between the expression of all genes and biomarkers were calculated. Genes were then ranked based on their correlation coefficients for GSEA on each biomarker, applying the criteria | Normalized Enrichment Score (NES)| > 1, p < 0.05, and False Discovery Rate (FDR) < 0.25. Single-gene GSEA for each biomarker was conducted using the ClusterProfiler package (v3.18.1) (Yu et al., 2012) in GSE151371. The top five most significant pathways in the Kyoto Encyclopedia of Genes and Genomes (KEGG), Gene Ontology (GO) Biological Process (BP), GO Cellular Component (CC), and GO Molecular Function (MF) for each biomarker were presented.

### 2.6 Immune infiltration analysis

For immune cell composition analysis, the GSE151371 samples were examined for immune infiltration using CIBERSORT, which provided the distributional proportions of various immune cell types. The proportions of immune cells between the control and SCI groups were compared using the Wilcoxon test (P < 0.05). The p-values were not corrected. The correlation of biomarkers with differential immune cell types was analyzed by Spearman correlation at P < 0.05 and |correlation (r)| > 0.3. The “HALLMARK_INFLAMMATORY_RESPONSE” was searched in the MSigDB database (https://www.gsea-msigdb.org/), and inflammation-related genes were obtained (Lu and Jia, 2022). The correlation between inflammation-related genes and differential immune cells was calculated through Spearman analysis (P < 0.05 and |correlation (r)| > 0.3). Genes were grouped, and the maximum absolute correlation was computed. The top 10 genes with the highest correlation were selected for display.

### 2.7 Clusters associated with histone lactonization modification

Based on the identified biomarkers, unsupervised consistent cluster analysis was performed on the 38 SCI samples from GSE151371 to determine distinct clusters. Principal component analysis (PCA) was then conducted to evaluate the differentiation ability of the clusters. Wilcoxon’s method was applied to assess differences in biomarker expression between the clusters.

### 2.8 Acquisition of DEGs

Differentially expressed genes (DEGs) between the clusters were identified using the limma package (version 3.56.2) (Ritchie et al., 2015), with criteria of adj. P < 0.05 and |log_2_foldchange (FC)| > 0.5. GO and KEGG analyses of these DEGs were performed using the ClusterProfiler package (P < 0.05) to explore the functional roles of the DEGs. The P-values were adjusted using the Benjamini–Hochberg (BH) correction method. In addition, the protein-protein interaction network diagram between the DEGs and HLMRGs was constructed using the Search Tool for the Retrieval of Interacting Genes (STRING) database (https://string-db.org/), showcasing the interactions between the DEGs and HLMRGs. The Confidence score was set to 0.9, the species was selected as “*Homo sapiens*,” and Cytoscape (v3.10.1) (Doncheva et al., 2019) was used for data visualization.

### 2.9 Functional exploration between clusters

Gene set variation analysis (GSVA) was employed to calculate the scores of Hallmark pathway gene sets using the GSVA package (v1.38.2) (Hanzelmann et al., 2013). Wilcoxon’s test was applied to compare the differences in pathway activity across clusters at P < 0.05. The p-values were not corrected. Additionally, differences in immune cell infiltration between the clusters were analyzed using Wilcoxon’s test (P < 0.05), and the correlation of differential immune cells with biomarkers was examined. The P-values were adjusted using the Benjamini–Hochberg (BH) correction method. Differences in immune function pathways and HLA genes (He et al., 2018) were also analyzed using Wilcoxon’s test, and the correlation between the 10 HLMRGs and immune function pathways or HLA genes was assessed by Spearman correlation analysis.

### 2.10 Identification of module genes and key proteins

To identify gene modules most strongly associated with the clusters, weighted gene co-expression network analysis (WGCNA) was performed using the WGCNA package (v1.72–1) (Langfelder and Horvath, 2008). Initially, all samples from different clusters were included in a sample clustering tree to remove outliers. A soft threshold (β) was determined based on the nearly scale-free topology criterion. The resulting topology matrix was clustered using gene differences (minModuleSize = 100, TOMType = “unsigned”, mergeCutHeight = 0.45, power = 16). The dynamic tree-cutting algorithm was used to partition the tree into modules, with different colors representing distinct gene modules and gray representing genes not assigned to any module. A heatmap of the relationships between module traits and clusters was plotted to evaluate the association of each module with different clusters. The most relevant module genes, with P < 0.05, were selected as key modules. Genes with |module membership (MM)| > 0.8 and |gene significance (GS)| > 0.2 were considered key module genes for constructing a protein-protein interaction (PPI) network. Protein interaction information was obtained from the Search Tool for the Retrieval of Interacting Genes (STRING) database (https://string-db.org/) to construct a PPI network. The Cytohubba plug-in (MCC, top = 10) was applied to identify the top 10 key proteins, which were recorded as key proteins for the clusters. The correlations between these 10 key proteins and biomarkers were analyzed using Spearman correlation analysis (P < 0.05 and |r| > 0.3).

### 2.11 Molecular networking

Transcription factors (TFs) for the biomarkers were predicted using the ChEA3 database. The miRNA interactions for the biomarkers were predicted using miRWalk (http://mirwalk.umm.uni-heidelberg.de/), Encyclopedia of RNA Interactome (ENOCRI, http://starbase.sysu.edu.cn/index.php), and miRTarBase (https://mirtarbase.cuhk.edu.cn/) databases. The common miRNAs identified through these databases were used to construct a miRNA-TF-biomarker interaction network. Additionally, potential drugs targeting the biomarkers were predicted using the Drug-Gene Interaction (DGI) database.

### 2.12 Expression validation of biomarkers

To validate the expression of biomarkers, SCI samples from GSE47681 were grouped separately into SCI and control categories for comparison. This study adhered to the principles outlined in the Declaration of Helsinki and was approved by the Ethics Committee of The Second Hospital of Anhui Medical University (Approval No. YW 2023-118, Date: 2023.07.06). All patients provided informed consent. To further verify the biomarker expression, reverse transcription-quantitative polymerase chain reaction (RT-qPCR) was performed on tissues from 5 SCI samples and five control samples. Clinical information of the patients is presented in Supplementary Table S1. RT-qPCR amplification was conducted for 40 cycles, consisting of 95°C for 1 min, 95°C for 20 s, 55°C for 20 s, and 72°C for 30 s. The primers used in RT-qPCR are shown in Table 1, with GAPDH as the reference gene. The relative expression levels of the biomarkers were calculated using the 2^−ΔΔCT^ method.

**TABLE 1:** List of primers for PCR.

genes	Primers
HDAC1 F	TGG​CCT​CTT​ACC​CAT​GTA​TCA​C
HDAC1 R	ATT​CTG​AGG​AGG​CAA​CAC​CG
HDAC2 F	GAG​CTT​TCG​GCA​CCT​CTG​C
HDAC2 R	GAA​ACG​TGG​GGG​CGA​TAG​TC
HDAC3 F	GAG​CAG​GGA​CTT​CAG​CCT​AC
HDAC3 R	GGG​ATT​GTG​TGA​ACG​CCA​AC
SIRT1 F	GGT​CGG​TGA​CAG​CCT​CAA​G
SIRT1 R	ATG​TCT​GCT​TCT​CCA​CCA​GC
SIRT3 F	GTA​GTT​GAA​CGG​GTC​GAG​GC
SIRT3 R	TAA​TAA​TCG​TCC​CTG​CCG​CC
LDHA F	TGC​CTT​GGG​CTT​GAG​CTT​TG
LDHA R	CAC​AGC​CAG​GCT​TCT​CAA​GT
LDHB F	GCC​TTC​TCT​CTC​CTG​TGC​AA
LDHB R	GAC​GGA​CTC​CTG​CAG​TTA​CC
GCN5 F	GAT​TGG​TCC​CTC​CTC​TCC​CT
GCN5 R	CCT​CTT​CTC​GCC​TGG​CAT​AG
GAPDH F	CGA​AGG​TGG​AGT​CAA​CGG​ATT​T
GAPDH R	ATG​GGT​GGA​ATC​ATA​TTG​GAA​C

### 2.13 Statistical analysis

All statistical analyses were performed using R software (v4.2.2). Differences between groups were assessed using Wilcoxon’s test, with P < 0.05 considered statistically significant.

## 3 Results

### 3.1 Eight DE-HLMRGs were significantly expressed between SCI and control samples

**FIGURE 1:**
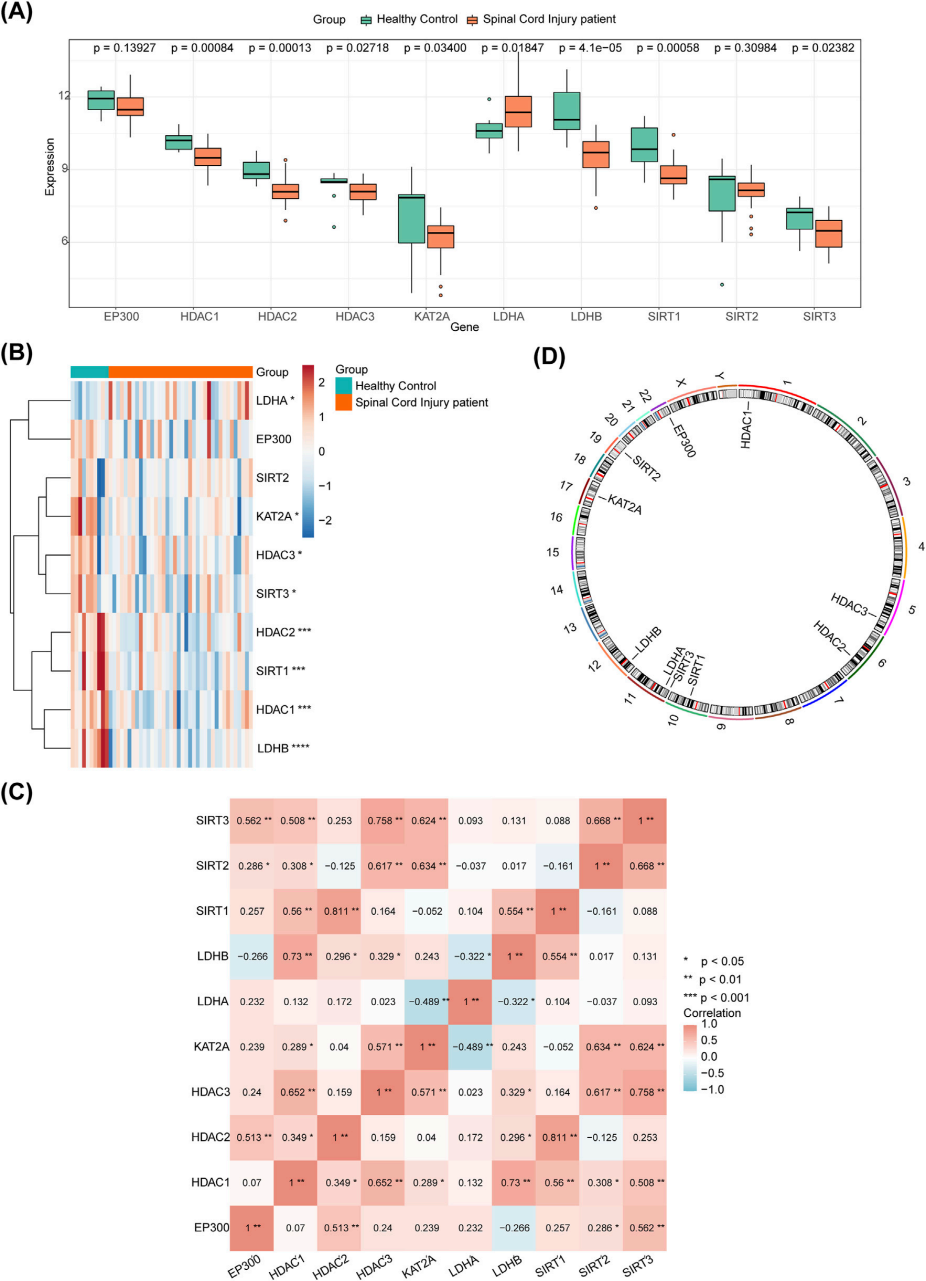
Expression analysis of DE-HLMRGs in SCI. **(A)** Expression of the 8 DE-HLMRGs in GSE151371. Horizontal coordinates represent HLMRGs, and vertical coordinates represent expression values. Asterisks indicate P-values, with more asterisks corresponding to more significant results; “ns” denotes not significant. **(B)** Heatmap showing the expression of 10 HLMRGs across two groups. Red represents high expression, while blue indicates low expression. **(C)** Correlation analysis of the 8 DE-HLMRGs. **(D)** Chromosome localization map for the 8 DE-HLMRGs.

In GSE151371, eight DE-HLMRGs exhibited significant expression differences between SCI and control samples (Figure 1A). These genes included *HDAC1*, *HDAC2*, *HDAC3*, *SIRT1*, *SIRT3*, LDHA, *LDHB*, and *GCN5* (*KAT2A*). *LDHA* was upregulated in SCI samples compared to controls, while the remaining genes were downregulated. The heatmap of the expression profiles of the 10 HLMRGs in both groups is shown in Figure 1B. Correlation analysis revealed significant positive correlations among most of the HLMRGs. The strongest positive correlation was observed between *HDAC2* and *SIRT1* (r = 0.811, P < 0.01), while the strongest negative correlation was found between *LDHA* and KAT2A (r = −0.489, P < 0.01) (Figure 1C). According to the chromosome localization map, *HDAC1* was located on chromosome 1, *HDAC3* on chromosome 5, and *HDAC2* on chromosome 6, among others (Figure 1D).

### 3.2 The logistic regression model had a great ability to discriminate control and SCI

**FIGURE 2:**
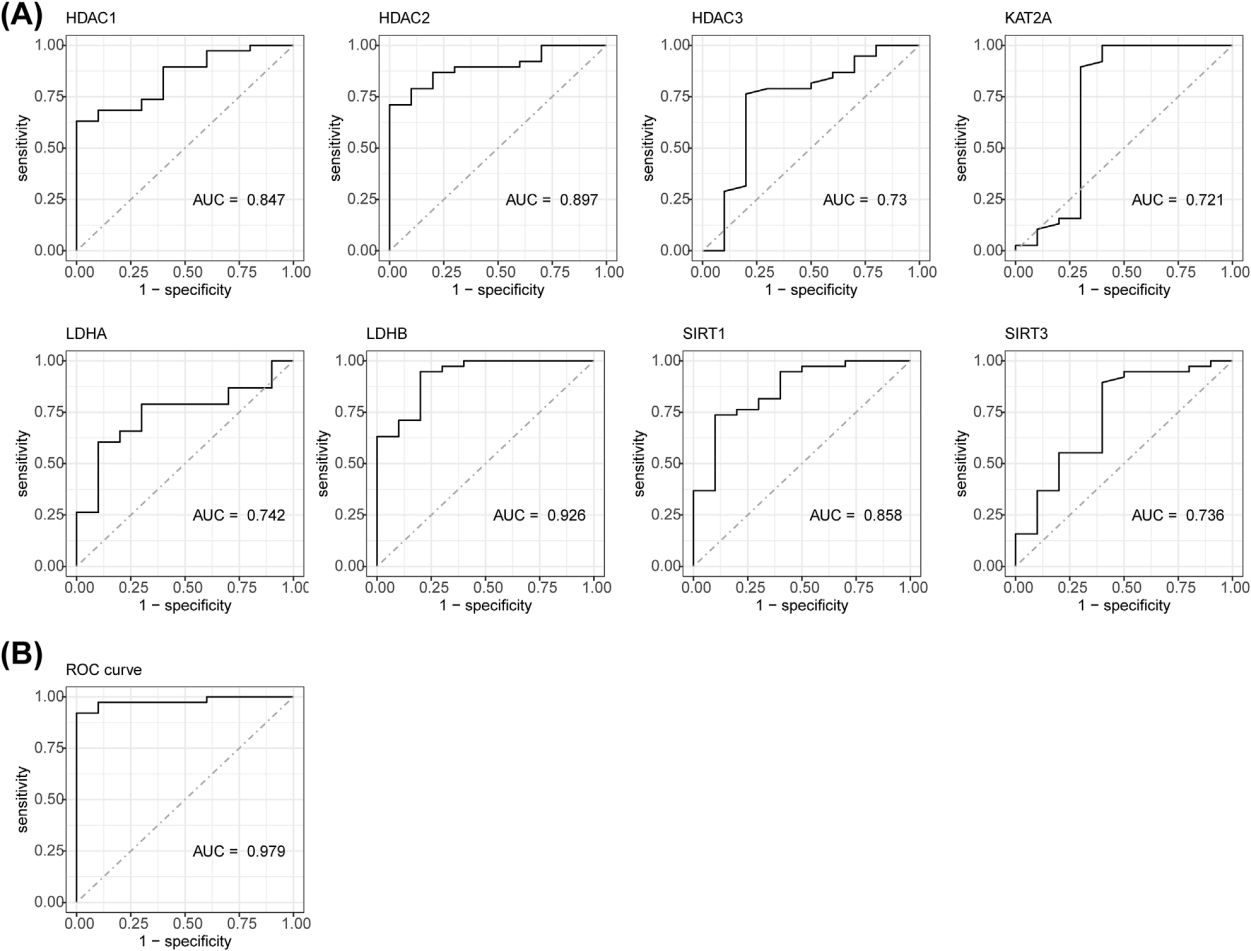
ROC curves for 8 DE-HLMRGs. **(A)** AUC values of ROC curves for the 8 DE-HLMRGs (biomarkers). **(B)** ROC curve for the logistic regression model.

The ROC curves for the eight DE-HLMRGs (biomarkers) showed AUC values greater than 0.7, indicating their potential to differentiate between SCI and control samples (Figure 2A). Furthermore, the ROC curve for the logistic regression model yielded an AUC of 0.979, suggesting excellent performance of the model (Figure 2B). The results from 10-fold cross-validation showed that the model exhibited excellent classification performance (AUC = 0.989) (Supplementary Figure S1).

### 3.3 The nomogram model had a great ability to predict SCI

**FIGURE 3:**
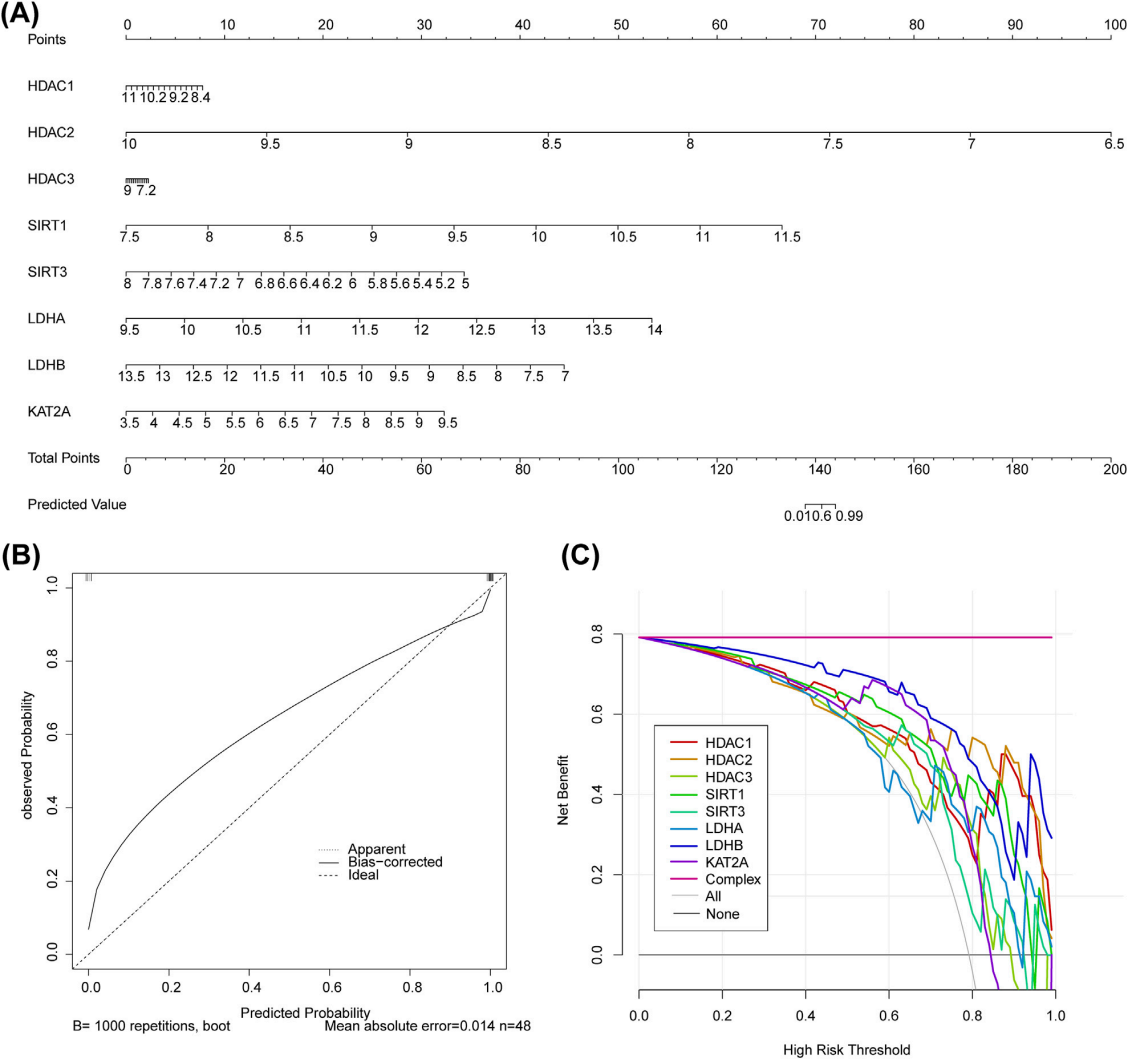
The biomarker-based nomogram model, calibration curve, and DCA curve. **(A)** Nomogram model constructed to predict SCI. **(B)** The C-statistic of the calibration curve is 0.987. **(C)** DCA curve.

A nomogram model was constructed based on the biomarkers from GSE151371 to predict SCI (Figure 3A). The calibration curve had a C-statistic of 0.987, indicating strong concordance between the apparent and predicted values, and suggesting the model’s high accuracy (Figure 3B). DCA demonstrated that the nomogram model offered a higher overall benefit to patients with SCI compared to individual biomarkers (Figure 3C).

### 3.4 Biomarkers were enriched in some metabolic pathways

**FIGURE 4:**
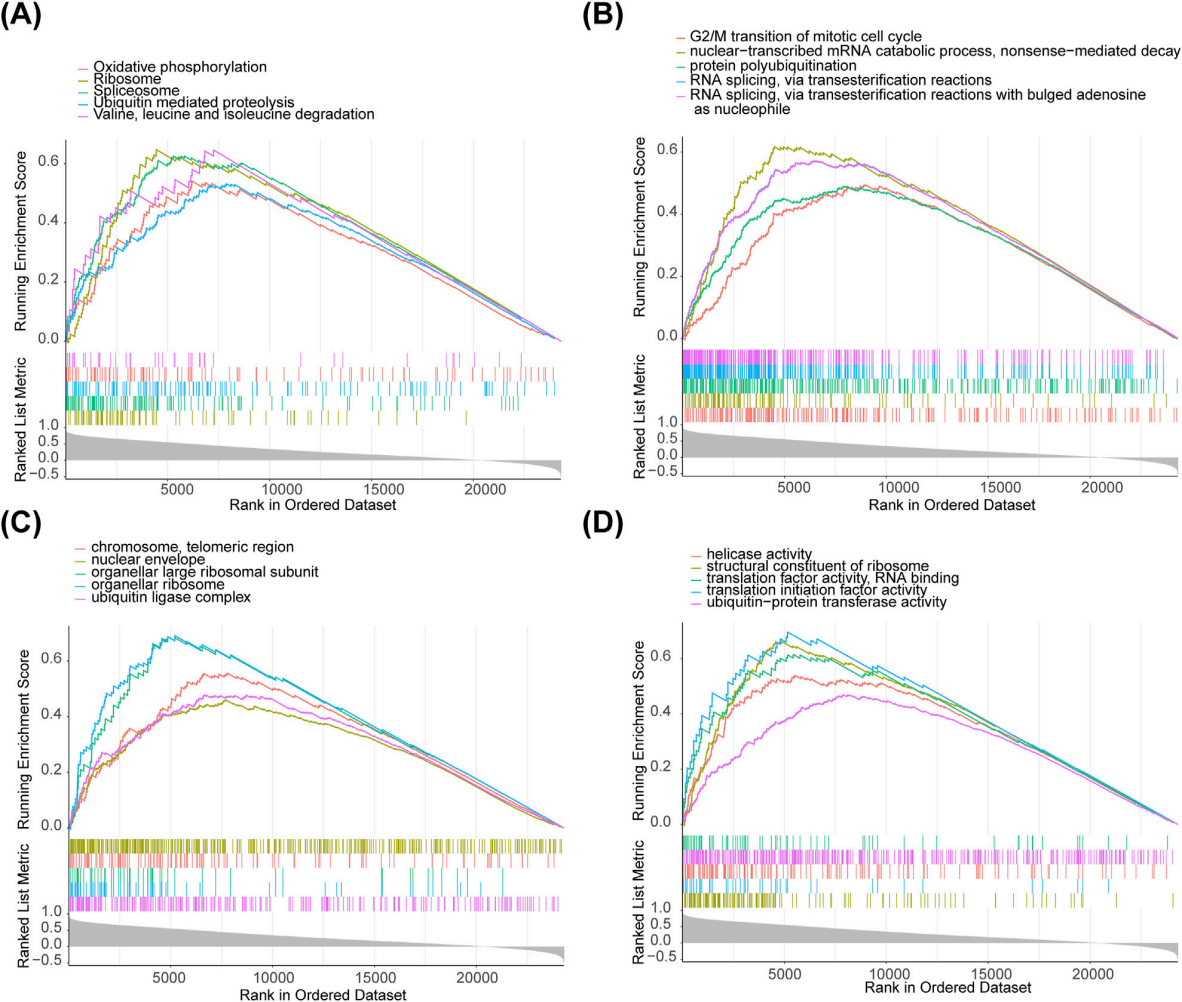
Biomarkers enriched in metabolic pathways. **(A)** HDAC1 enriched in 37 KEGG pathways. **(B)** 917 GO-BP pathways. **(C)** 209 GO-CC pathways. **(D)** 218 GO-MF pathways by GSEA.

For functional analysis, *HDAC1* was enriched in 37 KEGG pathways (Figure 4A), 917 GO-BP (Figure 4B), 209 GO-CC (Figure 4C), and 218 GO-MF (Figure 4D) by GSEA, including pathways such as ‘ubiquitin-mediated proteolysis’, ‘oxidative phosphorylation’, and ‘valine, leucine, and isoleucine degradation’. The enrichment results for the remaining seven biomarkers (*HDAC2*, *HDAC3*, *SIRT1*, *SIRT3*, *LDHA*, *LDHB*, and *GCN5* [*KAT2A*]) are shown in Supplementary Figures S2-8.

### 3.5 Fifteen immune cells differed in SCI and control groups

**FIGURE 5:**
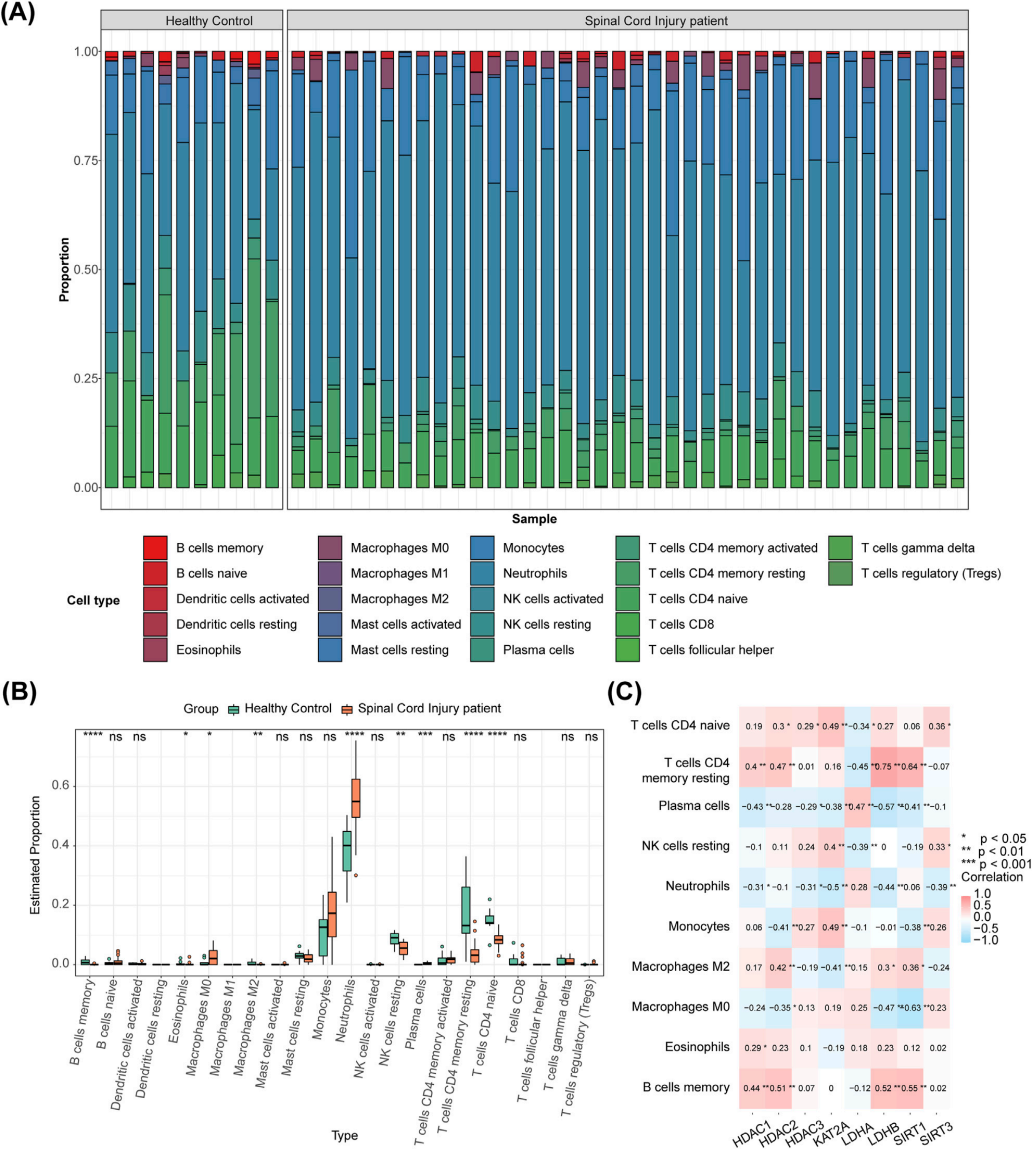
Analysis of immune cells with DE-HLMRGs. **(A)** The proportional distribution of immune cell types in each sample. **(B)** 15 immune cells differed between SCI and control groups. **(C)** Spearman correlation analysis showing the differential immune cells most significantly correlated with the biomarkers.

The immune cell composition in each sample is illustrated in Figure 5A. Between the SCI and control groups, 15 immune cell types showed significant differences, including memory B cells, eosinophils, and M0 macrophages (Figure 5B). Spearman correlation analysis revealed the differential immune cells most significantly associated with the biomarkers, including *HDAC1*-memory B cells (r = 0.440, P < 0.01), *HDAC2*-memory B cells (r = 0.510, P < 0.01), *HDAC3*-neutrophils (r = −0.310, P < 0.05), KAT2A-naive CD4 T cells/macrophages (both r = 0.490, P < 0.01), *LDHA*-plasma cells (r = 0.470, P < 0.01), *LDHB*-resting memory CD4 T cells (r = 0.750, P < 0.01), *SIRT1*-resting memory CD4 T cells (r = 0.640, P < 0.01), and *SIRT3*-neutrophils (r = −0.390, P < 0.01) (Figure 5C). Monocytes were significantly positively correlated with LPAR1 and MYC, while CD4 memory activated T cells were significantly negatively correlated with LPAR1 and MYC. The inflammatory factor IL7R was negatively correlated with T cells gamma delta and significantly positively correlated with activated dendritic cells (Supplementary Figure S9).

### 3.6 The two clusters exhibited distinguishing potential

**FIGURE 6:**
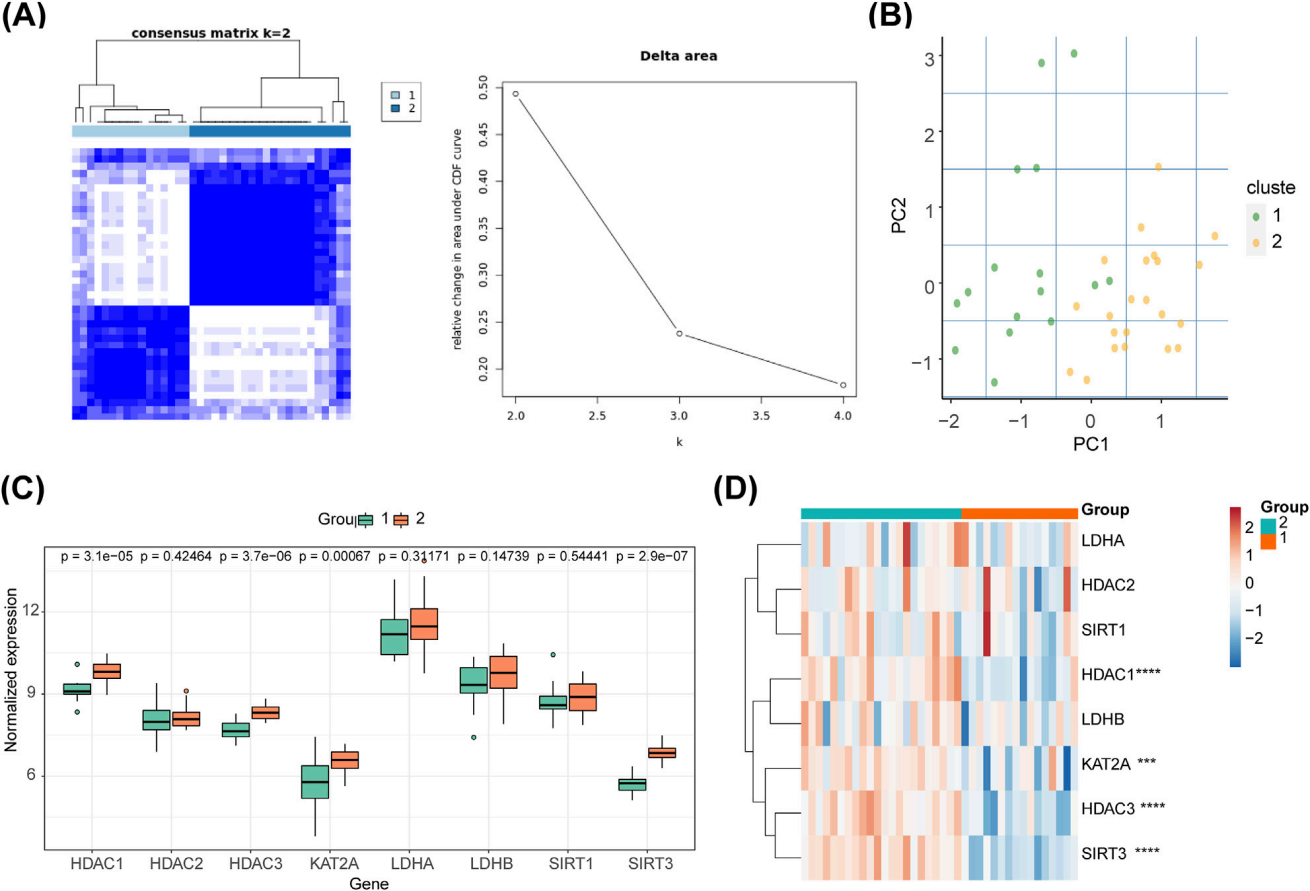
Consensus ClusterPlus analysis and PCA in different molecular patterns. **(A)** The 38 SCI samples from GSE151371 were classified into two clusters based on biomarkers. **(B)** PCA revealed differentiation between the two clusters. **(C)** Expression levels of all biomarkers across clusters. **(D)** Inter-cluster expression of the eight biomarkers displayed in a heatmap.

The 38 SCI samples from GSE151371 were categorized into two clusters based on biomarkers (Figure 6A). PCA indicated distinct differentiation between the two clusters (Figure 6B). Cluster two exhibited higher expression levels of all biomarkers, with significant differences observed only for *HDAC1*, *HDAC3*, *GCN5* (*KAT2A*), and *SIRT3* (P < 0.05) between the two groups (Figure 6C). The inter-cluster expression of the eight biomarkers is depicted in the heatmap (Figure 6D).

### 3.7 A total of 700 DEGs were enriched to 140 GO entries and 4 KEGG pathways

**FIGURE 7:**
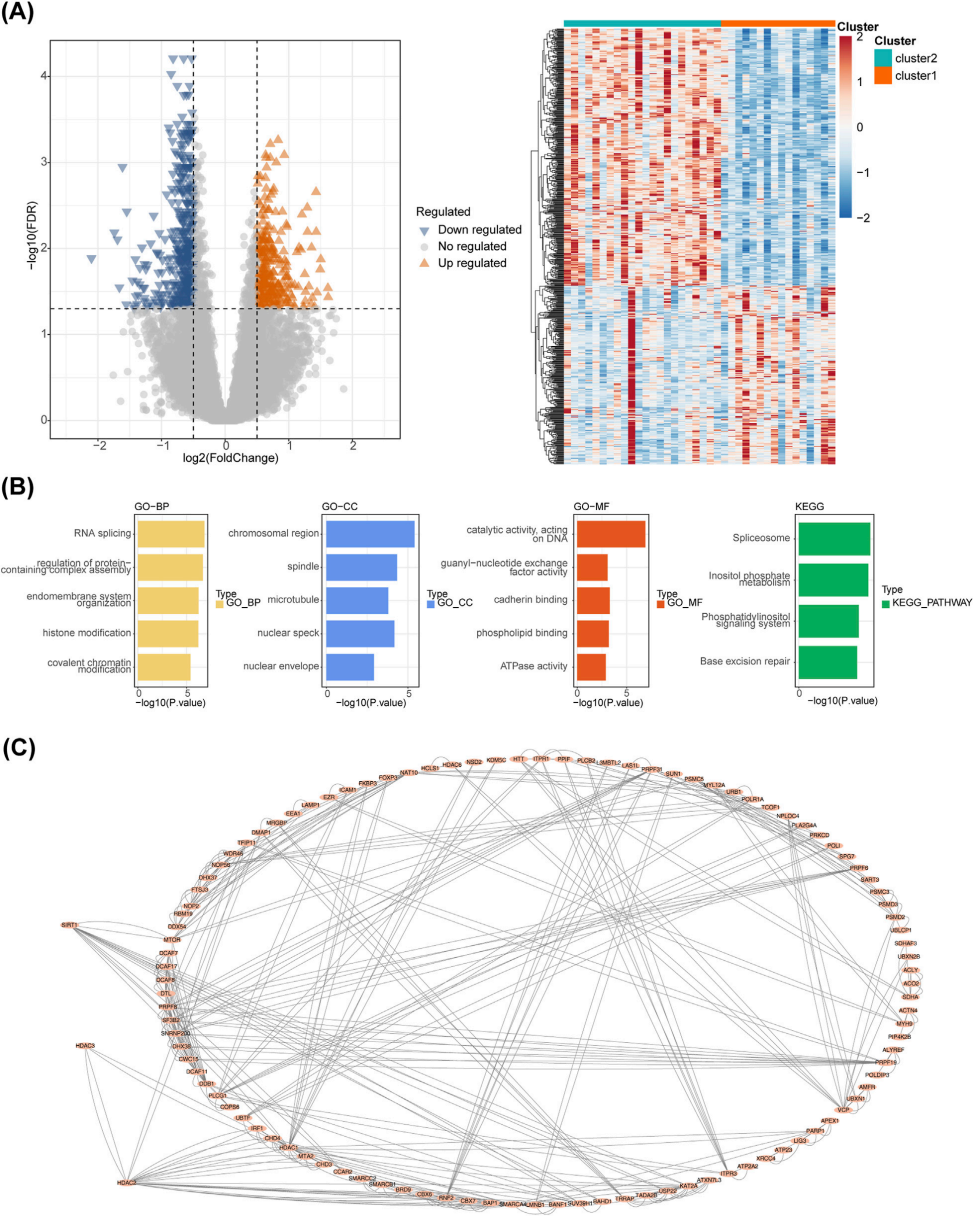
Analysis of differentially expressed genes. **(A)** Volcano plot and heatmap of differentially expressed genes between molecular patterns. **(B)** Enrichment analysis of differentially expressed genes between molecular patterns. **(C)** The PPI network diagram between the 700 DEGs and the eight HLMRGs.

A total of 700 DEGs were identified between clusters 1 and 2, including 287 upregulated and 413 downregulated genes (Figure 7A). These DEGs were enriched in 140 GO terms and four KEGG pathways, including ‘Base excision repair’, ‘phosphatidylinositol signaling system’, and ‘inositol phosphate metabolism’ (Figure 7B). In addition, the PPI network diagram between the 700 DEGs and the eight HLMRGs is shown in Figure 7C.

### 3.8 Hallmark pathways between clusters

**FIGURE 8:**
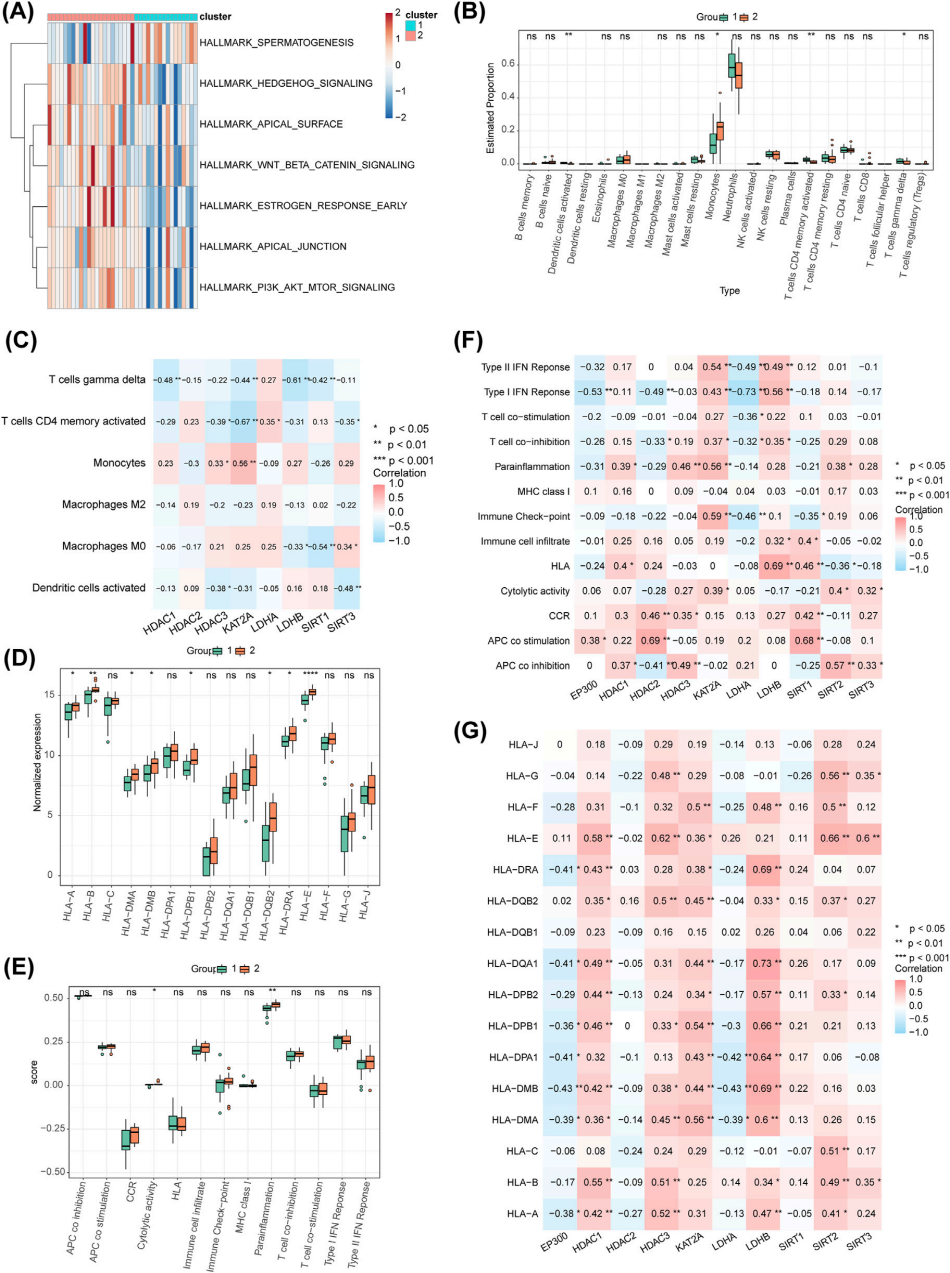
Hallmark pathway analysis, immune cell distribution, HLA genes, and immune function pathway score distribution. **(A)** Heatmap of differential Hallmark pathways between different molecular patterns. **(B)** Distribution of immune cells between different molecular patterns, showing only four immune cell types differing between clusters. **(C)** Analysis of the correlation between differential immune cells and biomarkers in different molecular patterns. **(D)** Distribution of immune function pathway scores across different molecular patterns. **(E)** Distribution of HLA genes across different molecular patterns. **(F)** Correlation analysis of functional pathway scores of the 10 HLMRGs. **(G)** Correlation analysis of HLA genes with the 10 HLMRGs.

Seven pathways showed significant differences between the clusters, including ‘apical junction’, ‘apical surface’, and ‘estrogen response early’ in the Hallmark set (Figure 8A). Four immune cell types, including activated dendritic cells, monocytes, activated CD4 memory T cells, and gamma delta T cells, displayed differential expression between the clusters (Figure 8B). Monocytes were positively correlated with *HDAC3* and KAT2A, while activated dendritic cells showed a significant negative correlation with *HDAC3* and *SIRT3* (Figure 8C). Differences in cytolytic activity and parainflammation were also observed between the clusters (Figure 8D). Expression differences of HLA genes, including HLA-A, HLA-B, HLA-DMA, HLA-DMB, HLA-DPB1, HLA-DQB2, HLA-DRA, and HLA-E, were noted across the clusters (Figure 8E). Regarding immune function, strong positive correlations were observed between *HDAC2* and APC co-stimulation (r = 0.690, P < 0.01), as well as between *LDHB* and HLA (r = 0.690, P < 0.01) (Figure 8F). Conversely, a strong negative correlation was found between *LDHA* and Type I IFN Response (r = −0.730, P < 0.01). The most significant positive correlation for HLA was observed between *LDHB* and HLA-DQA1 (r = 0.730, P < 0.01) (Figure 8G), while the strongest negative correlation was between *LDHA* and HLA-DMB (r = −0.430, P < 0.01).

### 3.9 Ten key proteins were selected

**FIGURE 9:**
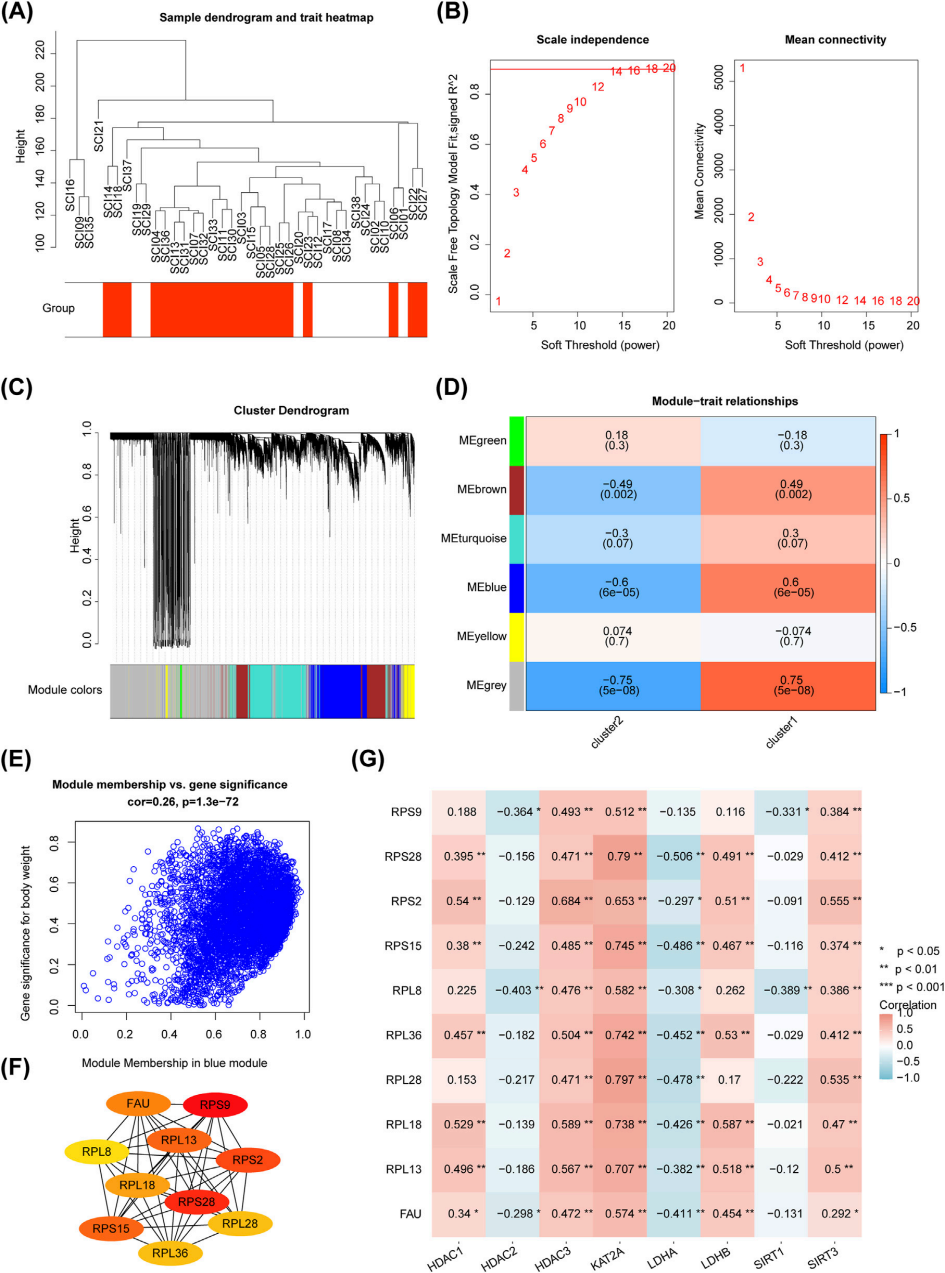
Screening of 10 key proteins. **(A)** Sample clustering dendrogram. **(B)** Squared correlation coefficients and network connectivity analysis. **(C)** Hierarchical clustering dendrogram for each module, with the top part showing the clustering dendrogram and the bottom part corresponding to the gene modules. Different colors represent distinct gene modules, and gray indicates genes that do not fit into any known module. **(D)** Correlation graph between modules and phenotypes. Modules with redder colors indicate a highly positive correlation with the phenotypic trait, while bluer colors indicate a strong negative correlation. **(E)** GS-MM plot. **(F)** Protein-protein interaction (PPI) network analysis for the 10 key proteins. **(G)** Correlation analysis between differential biomarkers and the 10 key genes.

For WGCNA, no outlier samples were identified within the clusters (Figure 9A). The soft threshold (β) was set to 16, which closely approximated a scale-free network (Figure 9B). Subsequently, six gene modules were identified through dynamic tree cutting (Figure 9C). Correlation analysis revealed that the blue module (r = −0.600, P < 0.05) was the most strongly associated with the clusters and was considered a key module (Figure 9D). A total of 1,615 module genes were selected for further analysis (Figure 9E). Based on the PPI network, 10 key proteins were identified: RPS9, RPS28, RPS2, RPS15, RPL13, FAU, RPL18, RPL36, RPL28, and RPL8 (Figure 9F). Correlation analysis showed that KAT2A (GCN5) was most strongly positively correlated with RPL28 (r = 0.797, P < 0.01), while *LDHA* showed the strongest negative correlation with RPL28 (r = −0.478, P < 0.01) (Figure 9G).

### 3.10 The miRNA-TF-biomarker and drug-biomarker networks

**FIGURE 10:**
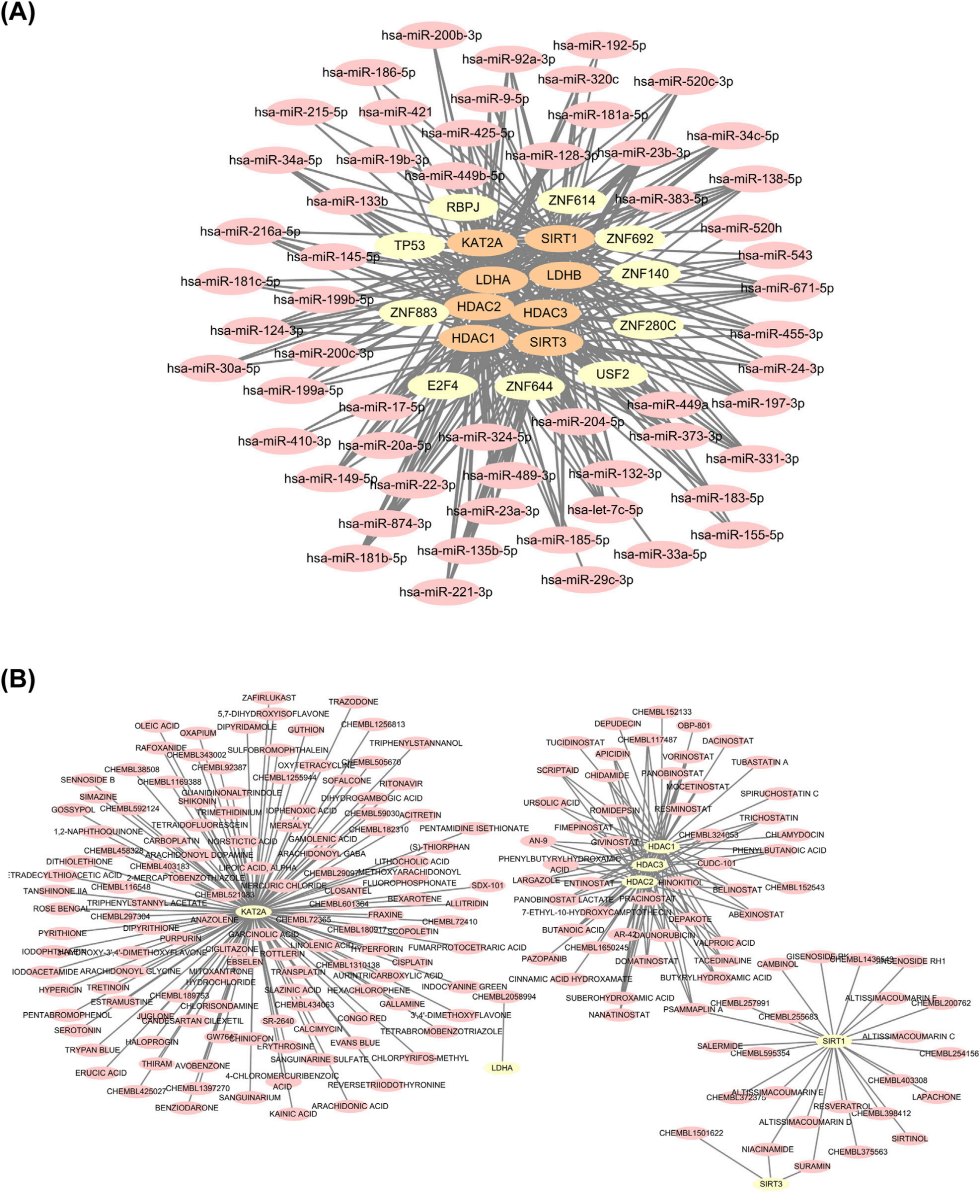
Analysis of miRNA/TF-biomarker interaction network and drug-biomarker interaction network. **(A)** miRNA/TF-biomarker interaction network. **(B)** Drug-biomarker interaction network.

A total of 41 TFs associated with the biomarkers were predicted using the ChEA3 database, from which the top 10 TFs were selected for network construction: ZNF883, ZNF614, ZNF644, ZNF280C, TP53, ZNF140, RBPJ, ZNF692, E2F4, and USF2. Additionally, 57 common miRNAs were predicted using miRWalk, ENOCRI, and miRTarBase databases. A miRNA-TF-biomarker interaction network was constructed based on the eight biomarkers, the top 10 TFs, and the 57 common miRNAs, revealing complex regulatory relationships (Figure 10A). Using the DGIdb database, 47 potential drugs were predicted for *HDAC1*, 39 for *HDAC2*, 38 for *HDAC3*, 24 for *SIRT1*, three for *SIRT3*, one for *LDHA*, and none for *LDHB*. *GCN5* (*KAT2A*) was predicted to interact with 126 potential drugs. A drug-biomarker interaction network comprising 279 edges and 208 nodes was created, including interactions such as niacinamide-*SIRT3*, suramin-*SIRT3*, and lapachone-*SIRT1* (Figure 10B).

### 3.11 *LDHA* was high expressed in SCI group, while *HDAC2*, *HDAC3*, *GCN5 (KAT2A)*, *LDHB*, and *SIRT3* were reversed

**FIGURE 11:**
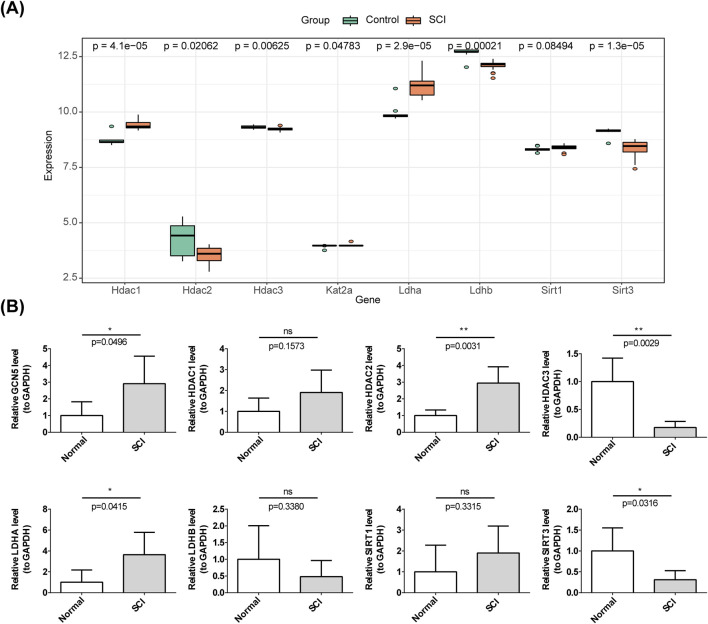
Validation of the HLMRGs. **(A)** Expression of the eight biomarkers in GSE47681. **(B)** qRT-PCR validation of the expression of the eight HLMRGs.

In the GSE151371 dataset (Figure 1A), only *LDHA* was highly expressed in the SCI group. In the GSE47681 dataset, *HDAC1*, *GCN5* (*KAT2A*), *LDHA*, and *SIRT1* were highly expressed in SCI samples, whereas *HDAC2*, *HDAC3*, *LDHB*, and *SIRT3* were downregulated in SCI samples (Figure 11A). RT-qPCR validation revealed that *HDAC1* (not significant), *HDAC2*, *GCN5* (*KAT2A*), *LDHA*, and *SIRT1* (not significant) were highly expressed, while *HDAC3*, *LDHB* (not significant), and *SIRT3* were downregulated in SCI samples (Figure 11B). Although *HDAC1*, *SIRT1*, and *LDHB* did not reach statistical significance, their expression trends were consistent with the results of the computational analysis in the dataset and require further validation. Among the eight biomarkers, *LDHA* exhibited high expression in SCI, while *HDAC3*, *LDHB*, and *SIRT3* were downregulated.

## 4 Discussion

SCI is a life-threatening and debilitating condition, recognized as a polygenic disorder influenced by alterations in numerous genes. The pathogenesis of SCI involves complex changes in multiple molecular pathways. In both SCI and brain injury, histone lactate plays a pivotal role in regulating various cellular processes, particularly in immune regulation and maintaining cellular homeostasis. This study utilized the GSE151371 and GSE47681 datasets to investigate 10 histone lactate-related genes (HLMRGs) and identified eight biomarkers (*HDAC1*, *HDAC2*, *HDAC3*, *SIRT1*, *SIRT3*, *LDHA*, *LDHB*, and *GCN5* [*KAT2A*]) based on ROC curve analysis. A comprehensive set of bioinformatics analyses was performed to explore potential new biomarkers and to elucidate the molecular mechanisms underlying SCI.

Our findings revealed expression differences in the 8 HLMRGs across various datasets and clinical blood samples, likely attributed to sample inconsistencies. Further validation through experiments will be necessary to confirm these results. The analysis of the validation set and tissue samples indicated that *LDHA* and *GCN5* (*KAT2A*) were highly expressed in SCI samples, while *HDAC3* and *SIRT3* exhibited reduced expression levels. In support of these findings, Guan C et al. (Guan and Wang, 2021) established SCI models through both *in vivo* and *in vitro* experiments, reporting elevated levels of *LDHA* and lactate in SCI rats and LPS-induced PC12 cells. *GCN5* (*KAT2A*) promotes axonal growth by regulating acetylated microtubule proteins (L et al., 2022), and loss of *GCN5* (*KAT2A*) activity induces neuronal apoptosis through the upregulation of Egr-1-dependent BH3-only protein Bim (Wu et al., 2017). These findings suggest that GCN5 (KAT2A) positively influences nerve injury recovery. In the present study, the upregulation of GCN5 (KAT2A) in SCI samples further supports the hypothesis that this gene is activated during SCI, contributing to injury repair. However, the precise mechanisms remain to be explored in future research. Additionally, our study observed low expression levels of *HDAC3* and *SIRT3* in SCI. Wahane (Wahane et al., 2021) reported that *HDAC3* activity regulates multiple transcriptional responses in SCI, particularly within myeloid and glial cells. Silencing or inhibiting *HDAC3* has been shown to improve neurological function and reduce spinal cord edema following SCI, suggesting its neuroprotective role (Dai et al., 2022; Zhou et al., 2020). In a rat model of SCI, downregulating *HDAC3* inhibited the activation of the JAK2/STAT3 pathway through the upregulation of miR-19b-1-5p, thereby promoting SCI recovery (Niu et al., 2022). The reduced expression of *HDAC3* and *SIRT3* in SCI may be part of a protective mechanism that promotes functional recovery by preventing Parthanatos and supporting antioxidant stress and mitochondrial function through *SIRT3*-mediated pathways (Jiang et al., 2023). Furthermore, activating the NMDAR/AMPK/PGC-1α/*SIRT3* signaling pathway through distal limb ischemic preconditioning has been shown to protect mice from spinal cord ischemia-reperfusion injury (Gu et al., 2023). Thus, *SIRT3* plays a significant role in SCI recovery and prognosis through multiple mechanisms. Clinically, SIRT3 inhibitors such as Suramin are already being explored for tumor treatment. In summary, *LDHA* and *SIRT3* may serve as effective biomarkers for diagnosing and treating SCI, offering novel insights into the molecular mechanisms and potential therapeutic pathways for SCI.

This study found that HLA genes, such as HLA-A, HLA-B, HLA-DMA, HLA-DMB and HLA-DPB1, show significant differences between Clusters one and 2. These genes are closely associated with antigen presentation (Álvaro-Benito and Freund, 2020; Di et al., 2021) and may influence the immune system’s ability to recognize and attack exogenous pathogens or tumor cells. Notably, the differences in HLA-DMA, HLA-DMB and HLA-DPB1 suggest different mechanisms of regulating immune responses. Specifically, LDHA shows a strong negative correlation with HLA-DMB, suggesting that LDHA may play a role in immune evasion or immune suppression by modulating specific pathways. Additionally, the negative correlation between LDHA and the type I interferon response indicates that LDHA may affect immune responses by inhibiting this response. This suggests that LDHA contributes to immune evasion. The negative correlation of LDHA with multiple immune functions further implies that it may be a key factor in immune evasion. Therefore, studying how LDHA regulates immune responses through metabolic pathways can help develop new inhibitors of immune evasion, thereby enhancing the efficacy of immunotherapy.

Our study conducted an enrichment analysis of 700 differentially expressed genes (DEGs) between molecular clusters one and 2, revealing that these genes are significantly associated with the phospholipid binding process. Previous studies demonstrated that spinal cord injury (SCI) could damage the integrity of the nerve fiber membrane at the nanoscale. The phospholipid binding domain, through its specific interaction with the phospholipid head group, can regulate the localization of membrane proteins and thereby influence the repair process of the spinal cord (Rad et al., 2015). Therefore, DEGs may play a potential regulatory role in the repair and functional recovery of the nerve membrane after SCI by participating in the phospholipid binding process; this involvement affects specific mechanisms of membrane repair following spinal cord injury. Furthermore, this study found through GSEA enrichment analysis that HDAC1 is significantly involved in the ubiquitin-mediated protein degradation and oxidative phosphorylation signaling pathways. Previous studies have shown that after SCI, the shift in oxidative phosphorylation in oligodendrocytes (OLs) leads to oxidative stress, which is further amplified by the upregulation of ferric protoporphyrin III (STEAP3). This finding suggests that oxidative phosphorylation plays a crucial role in the SCI process by regulating cell survival, death, and oxidative stress responses (Forston et al., 2023). Additionally, HDAC1, as a histone deacetylase, maintains the stability of mitochondrial oxidative phosphorylation and plays a key role in cellular energy metabolism and early development (Milstone et al., 2020). Therefore, after SCI, HDAC1 may regulate oligodendrocyte survival and mitochondrial function by modulating these key pathways, thereby exerting an important influence on the recovery process of SCI.

During SCI, multiple immune cell types play pivotal roles in both disease progression and recovery (Xu et al., 2021; Giron et al., 2023; Milich et al., 2019; Han et al., 2024). This study identified significant differences in four immune cell types: activated dendritic cells, monocytes, activated CD4 memory T cells, and gamma delta T cells. Previous research has shown that dendritic cells are influenced by *HDAC3* in various diseases, including AD and tumors (Han et al., 2022; Deng et al., 2019). Dendritic cells (DCs) are classified into plasmacytoid DCs (PDCs) and conventional DCs (CDCs). Both *in vivo* and *in vitro* experiments have demonstrated that histone deacetylase 3 (*HDAC3*), a key epigenetic regulator, is highly expressed in PDCs. The absence of *HDAC3* severely disrupts dendritic cell development (Zhang et al., 2023). In this study, a negative correlation between dendritic cells and *HDAC3* was observed, suggesting that multiple factors likely contribute to the regulation of dendritic cells in the SCI immune microenvironment, highlighting the need for further mechanistic investigations. *HDAC3* plays a key role in mediating the inflammatory response of human monocytes and macrophages, influencing their polarization, activation, and endotoxin tolerance in response to HDAC inhibitors (HDACIs) (Ghiboub et al., 2020). A positive correlation between monocytes and *HDAC3* in SCI was identified, suggesting new research avenues for understanding monocyte-driven inflammatory mechanisms in SCI. In studies of *HDAC3*-deficient mice, Eshleman (Eshleman et al., 2023) observed an increased accumulation of symbiotic-specific activated CD4 memory T cells in the gut, revealing a negative correlation between *HDAC3* and activated CD4 memory T cells in inflammatory diseases. A similar trend was seen in SCI in this study. Additionally, a negative correlation between activated CD4 memory T cells and *SIRT3* was observed, while a significant positive correlation with *LDHA* was noted, which aligns with previous findings in tumors and inflammatory diseases (Hou et al., 2024; Zou et al., 2024).

MicroRNAs (miRNAs) are small RNA molecules, approximately 22 nucleotides in length, that regulate protein expression by binding to specific sites on mRNA and inhibiting its translation. They are involved in various developmental pathways, gene regulatory mechanisms, and genetic disease processes, ultimately influencing phenotypic traits. Numerous miRNAs are present in the CNS, where they play critical roles in SCI progression (Silvestro and Mazzon, 2022). Modulating the NEAT1/miR-128-3p/AQP4 axis can alleviate neuropathic pain induced by SCI (Xian et al., 2021). Long non-coding RNA CASC9 (lncRNA CASC9) affects the levels of MDA, lactate, TNF-α, and IL-1β via miR-383-5p, thereby providing protection against oxidative stress, inflammation, and cell apoptosis in SCI (Guan and Wang, 2021). Moreover, silencing miR-324-5p alleviates *SIRT1*-induced SCI in rats (Wang et al., 2021). Based on the findings of this study, miRNAs are proposed as potential key factors in the progression of SCI.

This study identified and preliminarily validated biomarkers associated with histone lactylation in spinal cord injury (SCI), while also uncovering potential links between immune infiltrating cells and potential drug targets. These findings provide valuable insights for the further exploration of new biomarkers in SCI, potentially informing preventive and therapeutic strategies for SCI and offering a foundation for future research and clinical applications in this field. However, this study has limitations such as a small sample size, uneven distribution, and a lack of clinical information, which affect the generalizability and statistical reliability of the results. Although bioinformatics analysis suggests that HLMRGs have potential in the mechanisms and treatments of spinal cord injury (SCI), further validation is required through *in vitro* and *in vivo* experiments. Future research plans include expanding the sample size, adopting more robust statistical methods to enhance the reliability of the conclusions, and introducing Western blotting and immunofluorescence techniques to confirm changes in protein expression. Additionally, the efficacy and safety of the drugs will be evaluated using MTT and CCK-8 assays to assess cell viability and toxicity. Immunohistochemical staining will be used to observe the distribution of immune cells in the SCI injury area, revealing the relationship between immune cells and the pathological process. Moreover, apart from LDHA and LDHB, six other HLMRGs have been reported to exhibit strong functional conservation (Yang et al., 2022; Peng et al., 2024; Torres-Zelada and Weake, 2021; Soloviev et al., 2022). Due to physiological differences between humans and mice, cross-species validation may be affected. The current RT-qPCR results are preliminary, and future studies will require gene knockout, overexpression experiments, and more functional tests using animal models to further explore the biological functions of HLMRGs.

## Data Availability

The original contributions presented in the study are included in the article/Supplementary Material, further inquiries can be directed to the corresponding author.

## Abbreviations

SCI, Spinal Cord Injury; HLMRGs, Histone Lactylation Modification-Related Genes; GEO, Gene Expression Omnibus; DEGs, Differentially Expressed Genes; GSEA, Gene Set Enrichment Analysis; ROC, Receiver Operating Characteristic; AUC, Area Under the Curve; PCA, Principal Component Analysis; WGCNA, Weighted Gene Co-expression Network Analysis; PPI, Protein-Protein Interaction; TF, Transcription Factor; miRNA, microRNA; RT-qPCR, Reverse Transcription Quantitative Polymerase Chain Reaction; GO, Gene Ontology; KEGG, Kyoto Encyclopedia of Genes and Genomes; BP, Biological Process; CC, Cellular Component; MF, Molecular Function; GSVA, Gene Set Variation Analysis; DCA, Decision Curve Analysis; HLA, Human Leukocyte Antigen.
