# Supplementary material for: Exploration of biomarkers associated with histone lactylation modification in spinal cord injury
Source: Front Genet. 2025 Jul 2;16:1609439. doi: 10.3389/fgene.2025.1609439 (PMC12263368; doi:10.3389/fgene.2025.1609439)
Supplement: Supplementary file 1 [file DataSheet1.pdf]

Supplementary Material

1 Supplementary Figures

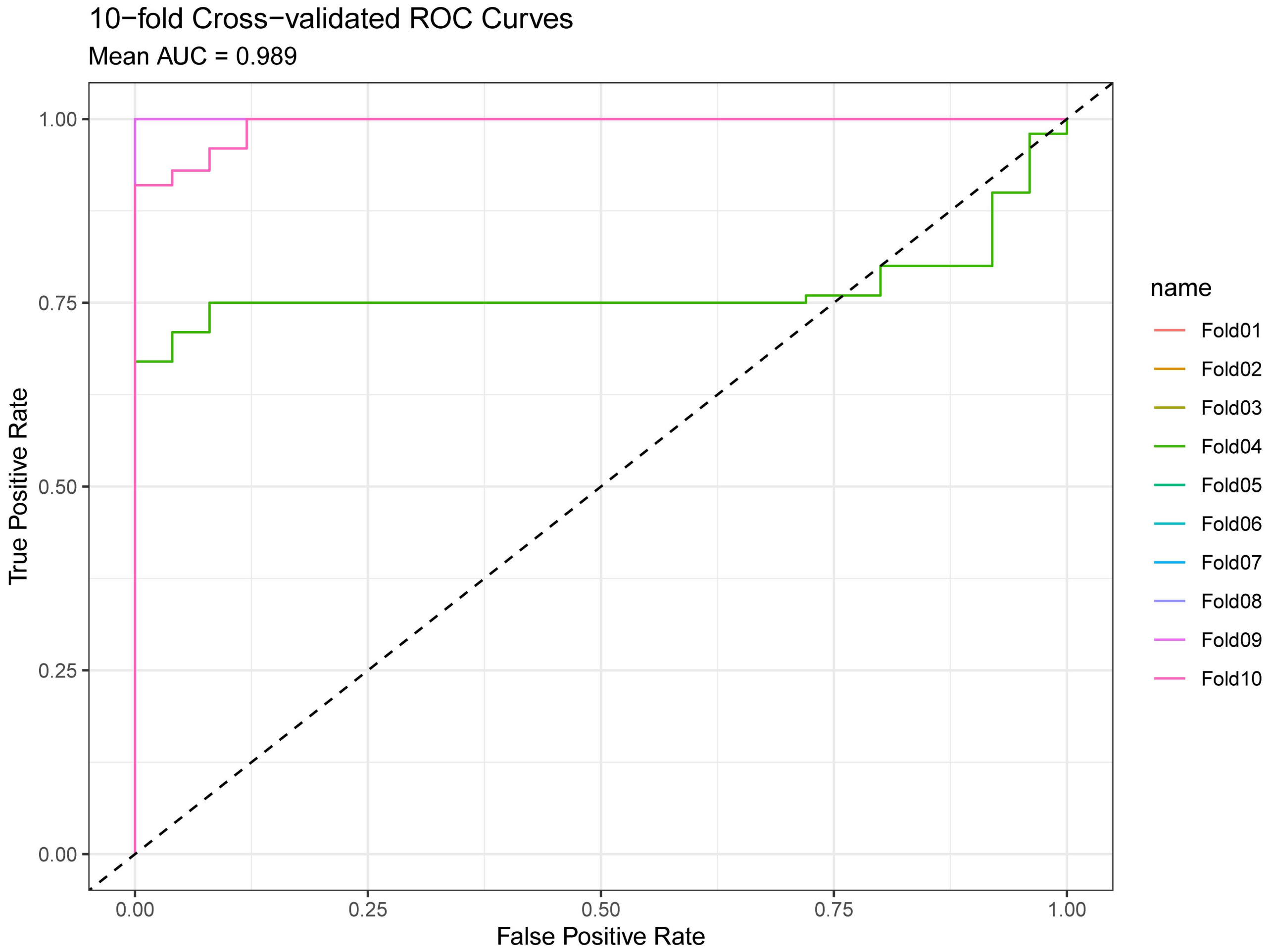

Supplementary Figure 1. The results of 10-fold cross-validation.

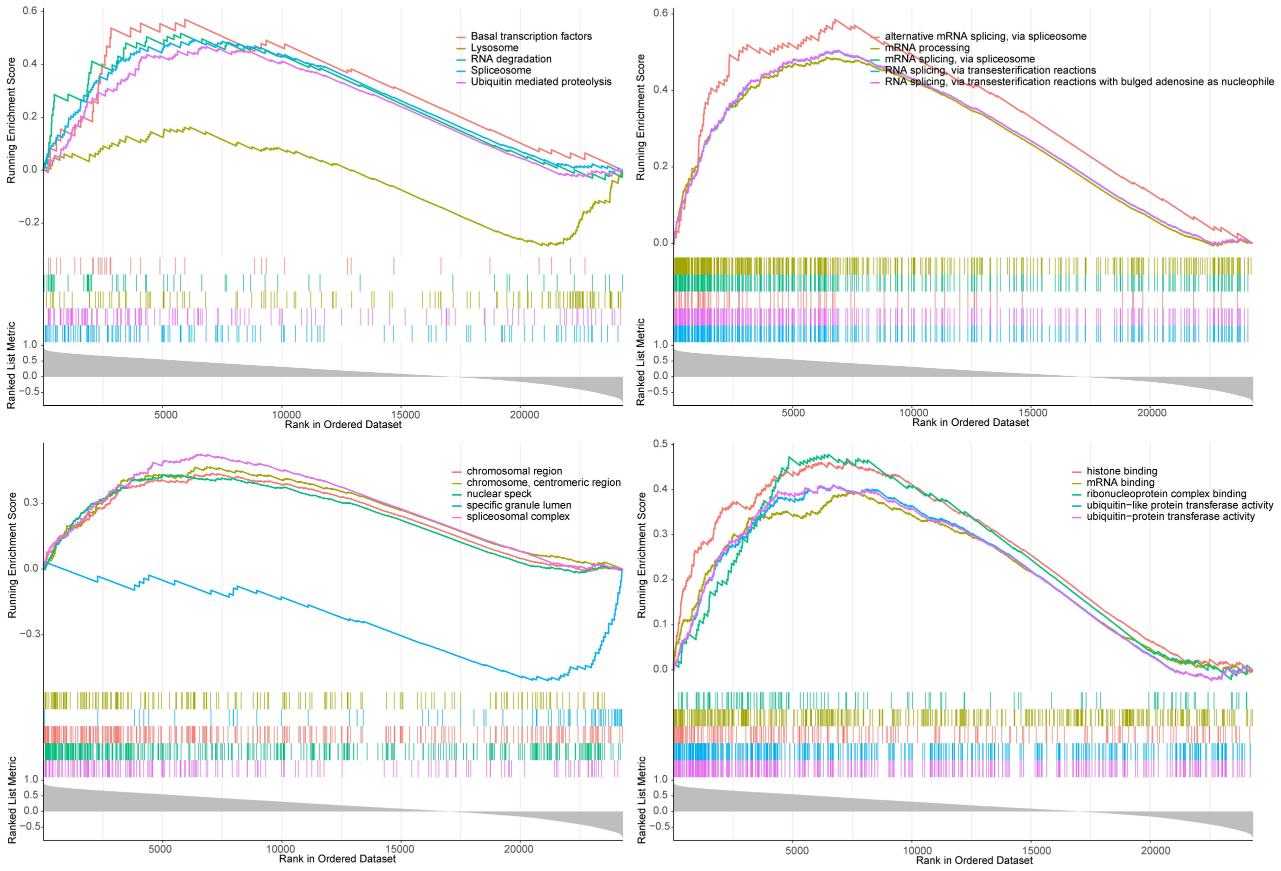

Supplementary Figure 2. Results of KEGG and GO enrichment analysis of HDAC2.

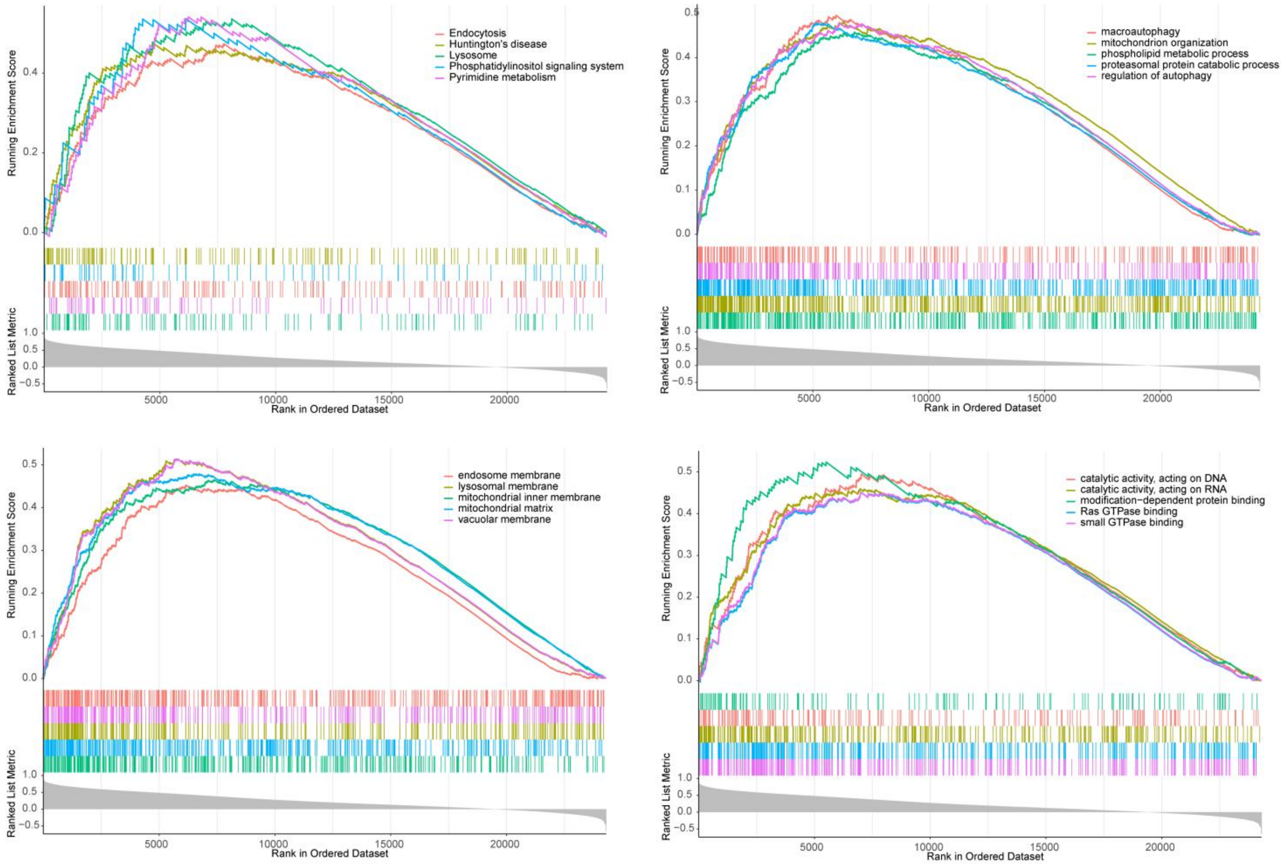

Supplementary Figure 3. Results of KEGG and GO enrichment analysis of HDAC3.

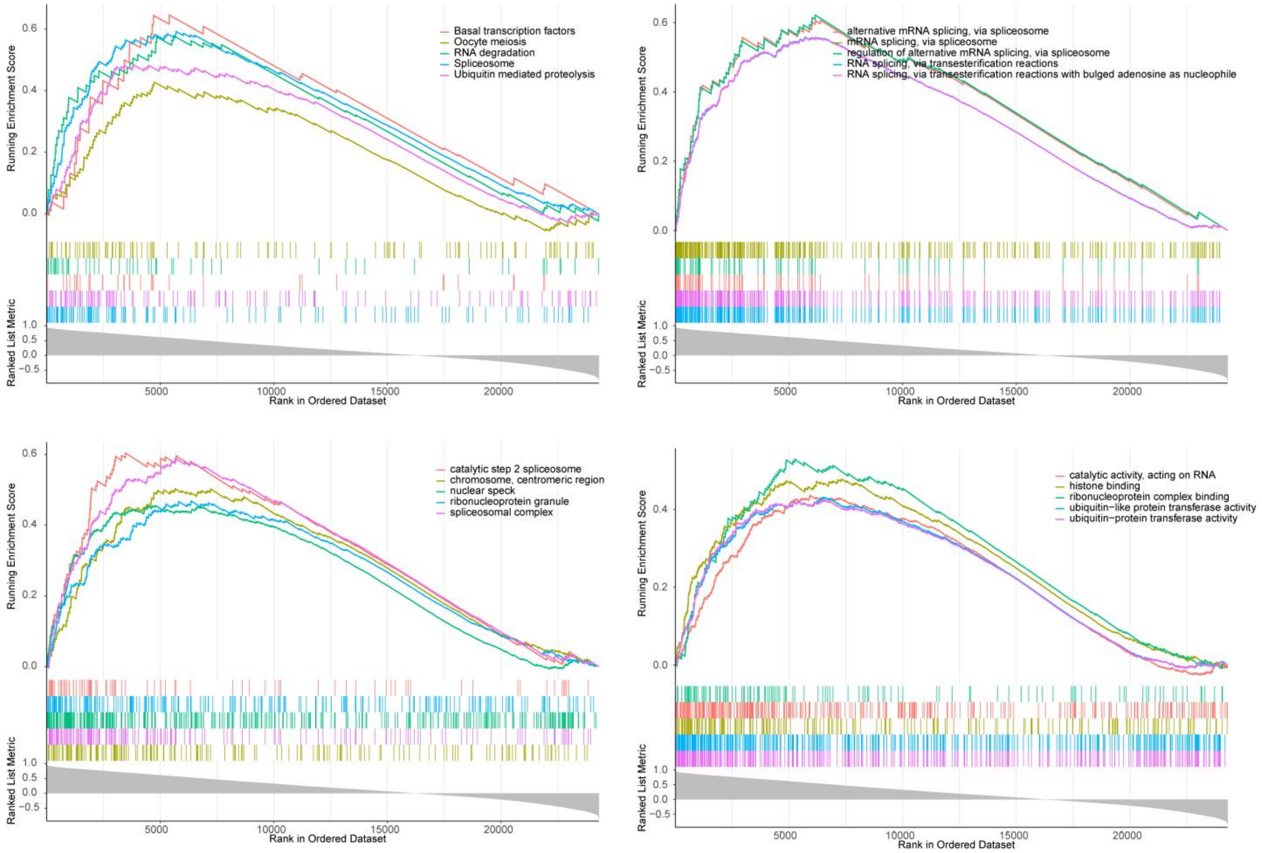

Supplementary Figure 4. Results of KEGG and GO enrichment analysis of SIRT1.

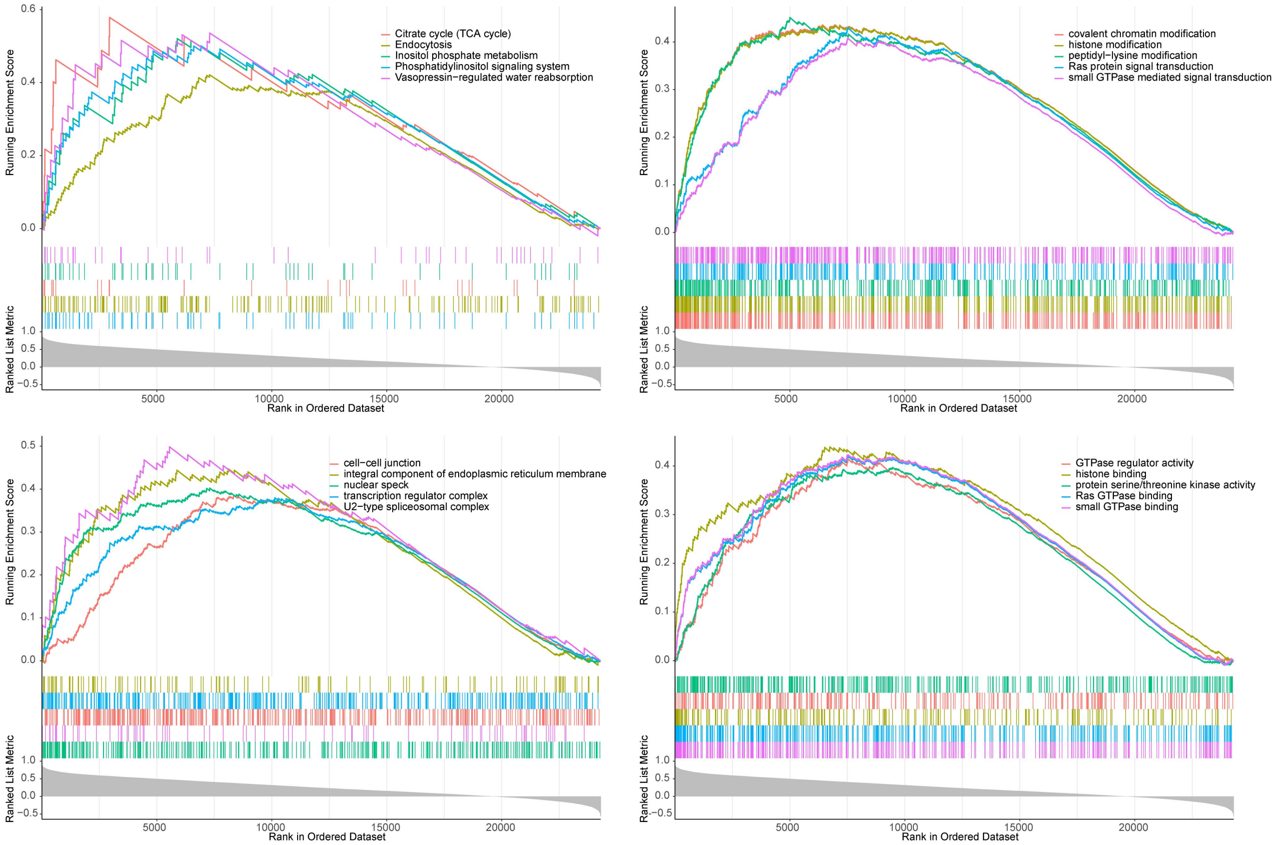

Supplementary Figure 5. Results of KEGG and GO enrichment analysis of SIRT3.

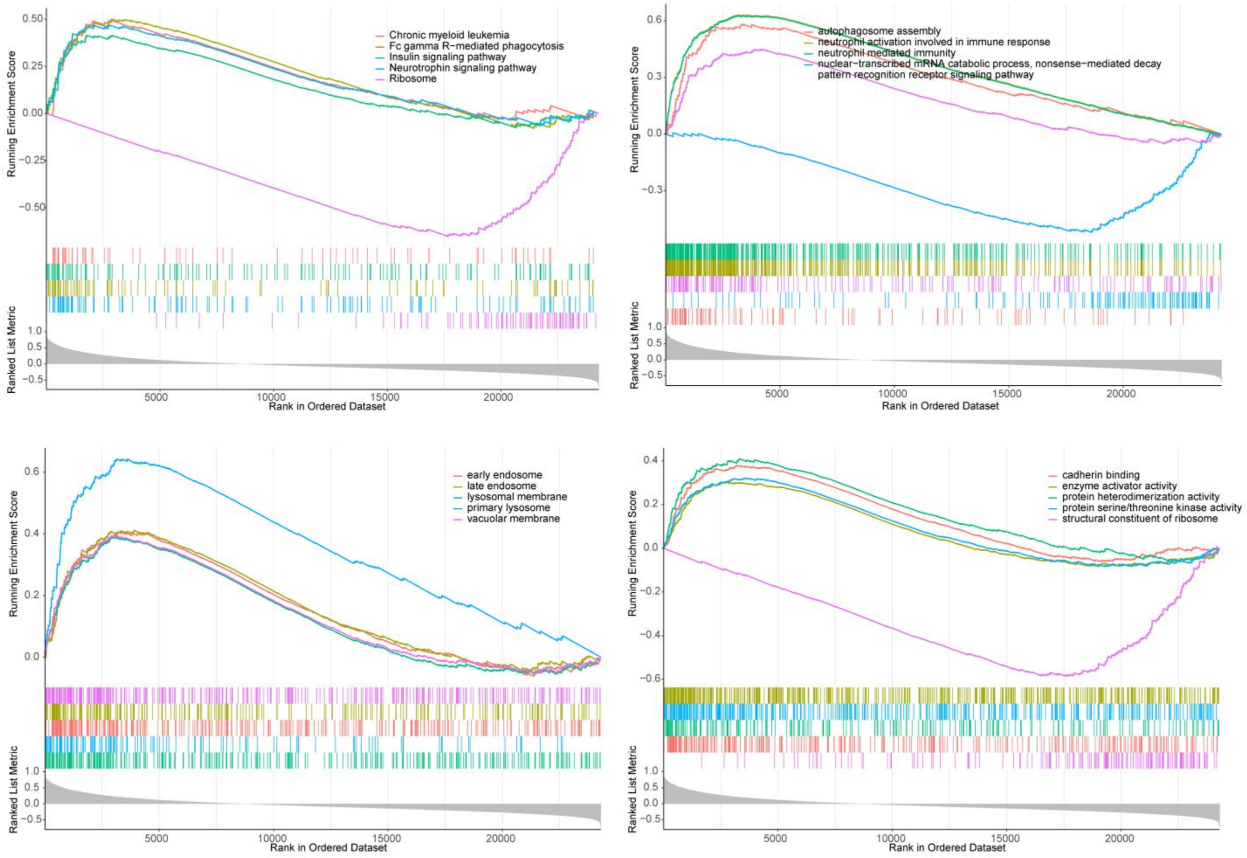

Supplementary Figure 6. Results of KEGG and GO enrichment analysis of LDHA.

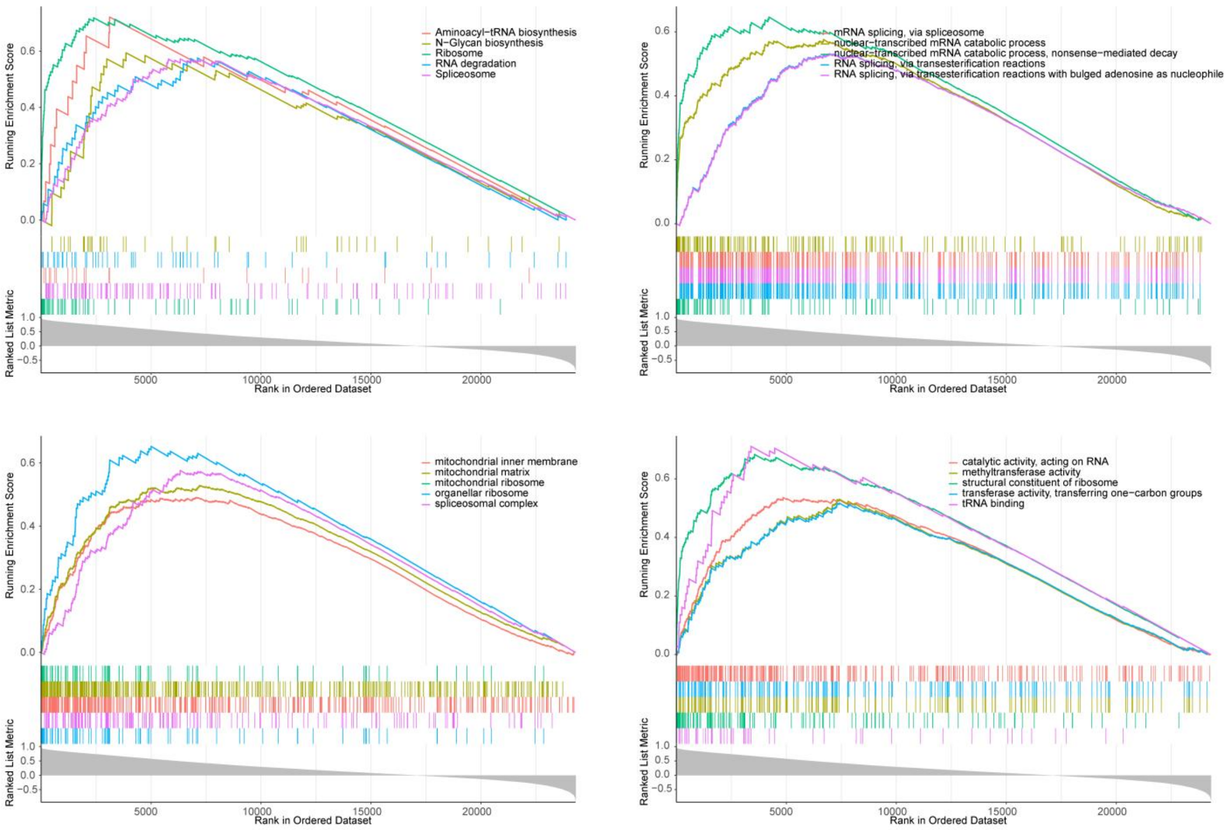

Supplementary Figure 7. Results of KEGG and GO enrichment analysis of LDHB.

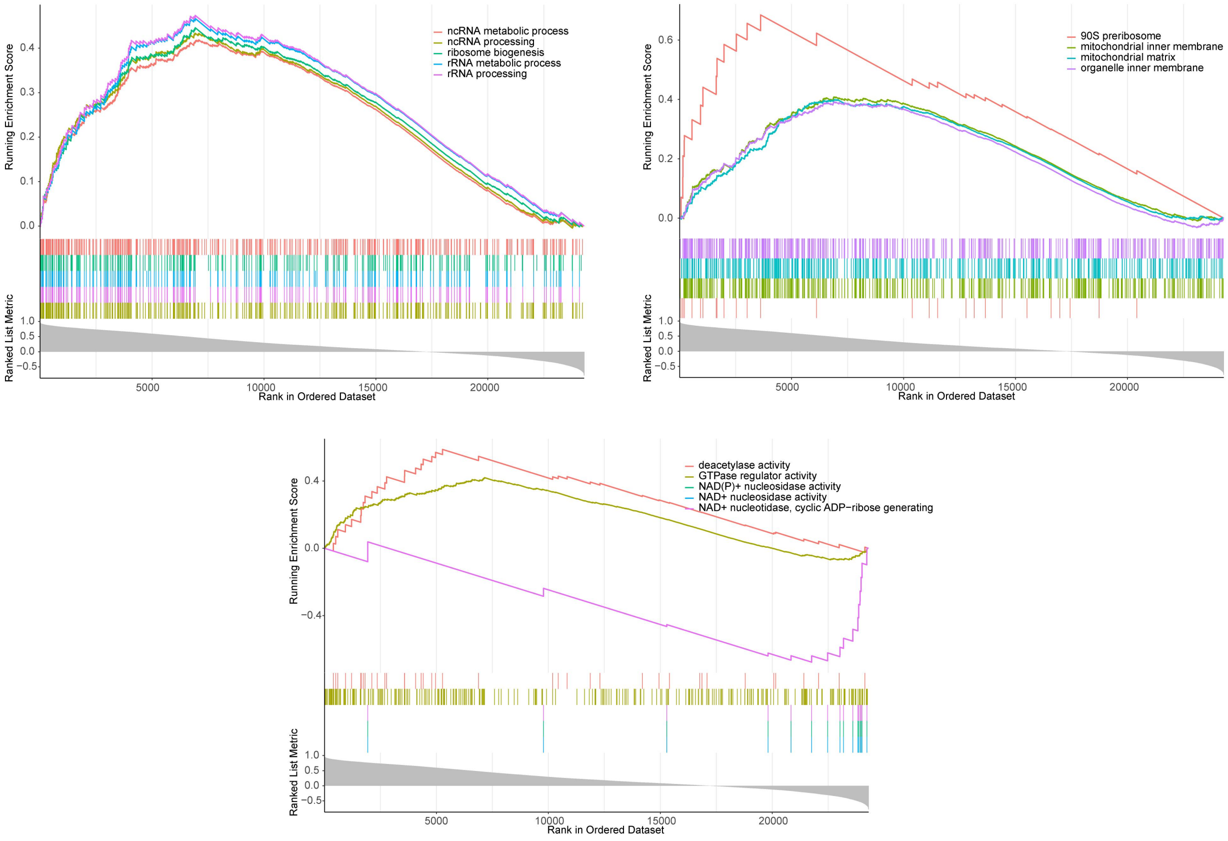

Supplementary Figure 8. Results of KEGG and GO enrichment analysis of GCN5 (KAT2A).

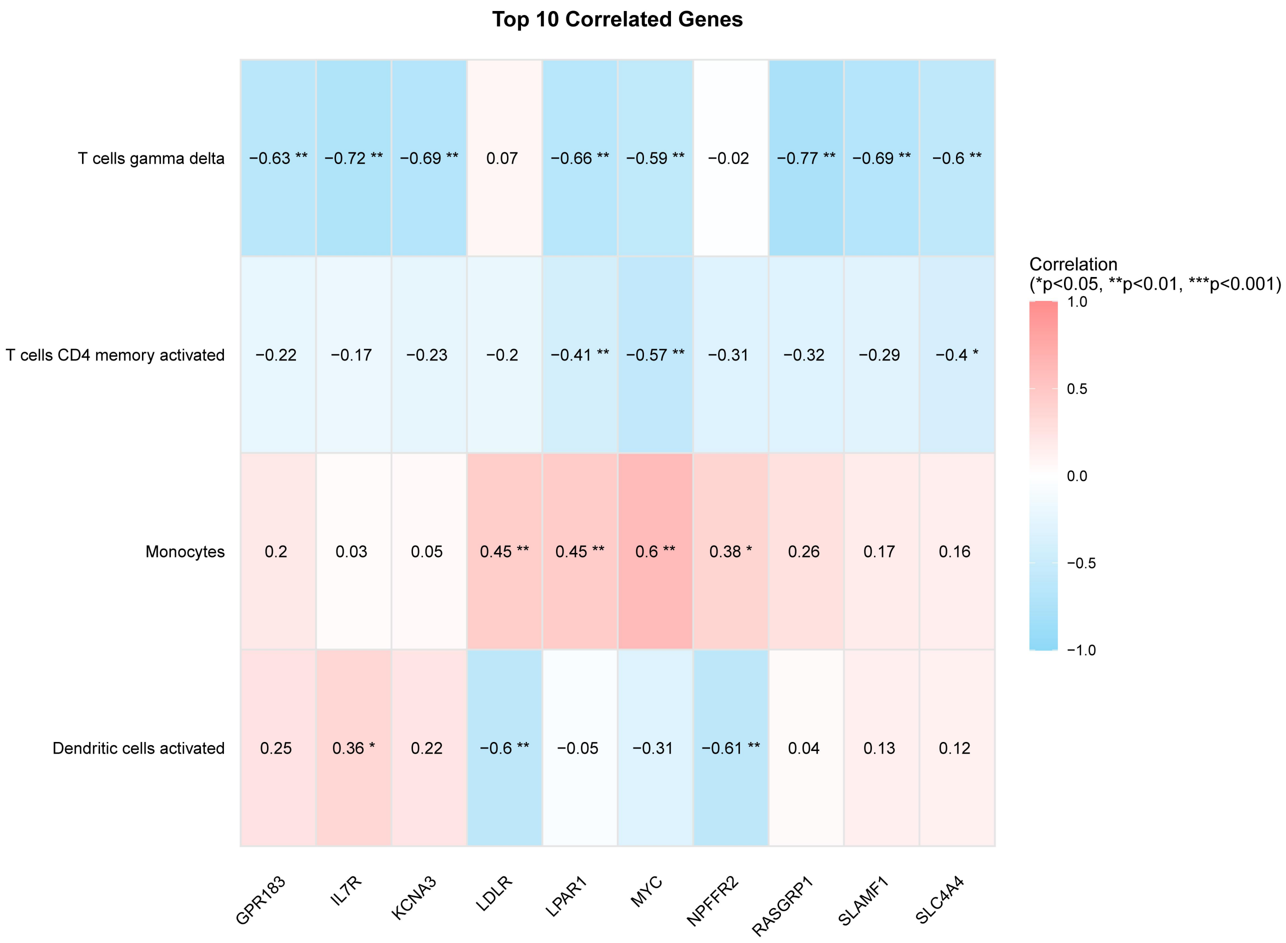

Supplementary Figure 9. The correlation between differential immune cells and related inflammatory factors.

2 Supplementary Tables

Supplementary Table 1 Clinical information on the patients
